# Chronic kidney disease promotes atrial fibrillation via inflammasome pathway activation

**DOI:** 10.1172/JCI167517

**Published:** 2023-10-02

**Authors:** Jia Song, Jose Alberto Navarro-Garcia, Jiao Wu, Arnela Saljic, Issam Abu-Taha, Luge Li, Satadru K. Lahiri, Joshua A. Keefe, Yuriana Aguilar-Sanchez, Oliver M. Moore, Yue Yuan, Xiaolei Wang, Markus Kamler, William E. Mitch, Gema Ruiz-Hurtado, Zhaoyong Hu, Sandhya S. Thomas, Dobromir Dobrev, Xander H.T. Wehrens, Na Li

**Affiliations:** 1 Department of Medicine (Cardiovascular Research),; 2 Cardiovascular Research Institute,; 3 Department of Integrative Physiology, and; 4 Selzman Institute for Kidney Health, Department of Medicine, Baylor College of Medicine, Houston, Texas, USA.; 5 Institute of Pharmacology, University Duisburg–Essen, Essen, Germany.; 6 Department of Biomedical Sciences, Faculty of Health and Medical Sciences, University of Copenhagen, Copenhagen, Denmark.; 7 Department of Thoracic and Cardiovascular Surgery, West German Heart and Vascular Center, University Duisburg–Essen, Essen, Germany.; 8 Cardiorenal Translational Laboratory, Institute of Research Imas12, Hospital Universitario 12 de Octubre, Madrid, Spain.; 9 CIBER-CV, Hospital Universitario 12 de Octubre, Madrid, Spain.; 10 Department of Medicine (Nephrology Division), Baylor College of Medicine, Houston, Texas, USA.; 11 Department of Medicine and Research Center, Montreal Heart Institute and Université de Montréal, Montréal, Canada.; 12 Department of Medicine (Cardiology),; 13 Department of Neuroscience,; 14 Department of Pediatrics (Cardiology),; 15 Center for Space Medicine, Baylor College of Medicine, Houston, Texas, USA.

**Keywords:** Cardiology, Arrhythmias, Chronic kidney disease

## Abstract

Chronic kidney disease (CKD) is associated with a higher risk of atrial fibrillation (AF). The mechanistic link between CKD and AF remains elusive. IL-1β, a main effector of NLR family pyrin domain–containing 3 (NLRP3) inflammasome activation, is a key modulator of conditions associated with inflammation, such as AF and CKD. Circulating IL-1β levels were elevated in patients with CKD who had AF (versus patients with CKD in sinus rhythm). Moreover, NLRP3 activity was enhanced in atria of patients with CKD. To elucidate the role of NLRP3/IL-1β signaling in the pathogenesis of CKD-induced AF, *Nlrp3^–/–^* and WT mice were subjected to a 2-stage subtotal nephrectomy protocol to induce CKD. Four weeks after surgery, IL-1β levels in serum and atrial tissue were increased in WT CKD (WT-CKD) mice versus sham-operated WT (WT-sham) mice. The increased susceptibility to pacing-induced AF and the longer AF duration in WT-CKD mice were associated with an abbreviated atrial effective refractory period, enlarged atria, and atrial fibrosis. Genetic inhibition of NLRP3 in *Nlrp3^–/–^* mice or neutralizing anti–IL-1β antibodies effectively reduced IL-1β levels, normalized left atrial dimensions, and reduced fibrosis and the incidence of AF. These data suggest that CKD creates a substrate for AF development by activating the NLRP3 inflammasome in atria, which is associated with structural and electrical remodeling. Neutralizing IL-1β antibodies may be beneficial in preventing CKD-induced AF.

## Graphical abstract

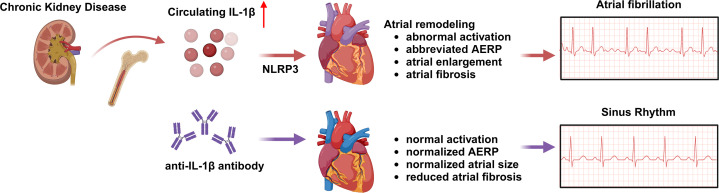

## Introduction

Atrial fibrillation (AF) is the most commonly diagnosed cardiac arrhythmia, with a high prevalence worldwide (1, 2). AF significantly affects the quality of life, as it is associated with an increased risk of heart failure and stroke (3, 4). The incidence and prevalence of AF are predicted to increase as a result of the aging population and the rising occurrence of other risk factors (5). The burden of AF is even higher in patients with chronic kidney disease (CKD), who have a 3-fold increased risk of AF compared with healthy individuals (5, 6). The treatment of patients with CKD continues to be challenging (7), given the poor understanding of the molecular mechanisms that cause AF in CKD (8).

Patients with CKD commonly exhibit chronic systemic inflammation syndrome (9), characterized by increased levels of IL-1β, IL-6, and C-reactive protein (CRP) (10, 11). This inflammatory condition may play an important role in the increased morbidity and mortality associated with CKD. Dysregulated mineral metabolism and increased oxidative stress are thought to cause the proinflammatory conditions seen in patients with CKD (12). It has also been suggested that inflammation-driven activation of the innate immune system contributes to cardiac dysfunction in the context of CKD (13). The innate immune system is activated through pattern recognition receptors (PRRs) that recognize pathogen-associated molecular patterns (PAMPs) or danger-associated molecular patterns (DAMPs). PRRs of the innate immune system are thought to initiate the host response after kidney (14) and cardiac (15, 16) damage. The most common PRRs expressed in the heart are TLRs, nucleotide-binding oligomerization domain–like (NOD-like) receptors (NLRs), RIG-I-like receptors, absent in myeloma 2–like (AIM2-like) receptors (ALRs), and C-type lectin receptors (CLRs) (17). NLRs are cytosolic receptors activated by both PAMPs and DAMPs that lead to inflammatory responses by the assembly of caspase-1 with subsequent activation of IL-1β and IL-18 (14, 18). NLRs associate with the adaptor protein apoptosis-associated speck-like protein containing a CARD (ASC) and pro–caspase-1, forming the inflammasome (19). It has been reported that the levels of NLR family pyrin domain–containing 3 (NLRP3) inflammasome and other inflammasome-related genes are upregulated in human kidney diseases. NLRP3 has been proposed as a potential target to treat experimental CKD (20, 21). Interestingly, recent studies have reported that cardiac expression of NLRP3 is increased in experimental models of CKD (22, 23), suggesting a potential role for the NLRP3 inflammasome in kidney disease–induced cardiac dysfunction. On the other hand, recent studies demonstrated that increased cardiac NLRP3 inflammasome activity is associated with adverse cardiac remodeling (24) and dysfunction (25). Emerging evidence indicates that the NLRP3 inflammasome activation contributes to the onset and progression of AF (26). Cardiac NLRP3 expression was shown to be increased in atrial samples from patients with AF (27, 28) and in animals models that develop spontaneous AF (29). However, whether CKD-induced AF development is causally linked to NLRP3 activation has not been studied. Here, we demonstrate that genetic ablation of NLRP3 and antibodies neutralizing circulating IL-1β can effectively prevent atrial remodeling and the development of an AF-promoting substrate, both of which are associated with the development of CKD.

## Results

### Enhanced activity of NLRP3 inflammasomes in CKD patients with AF.

**Figure 1:**
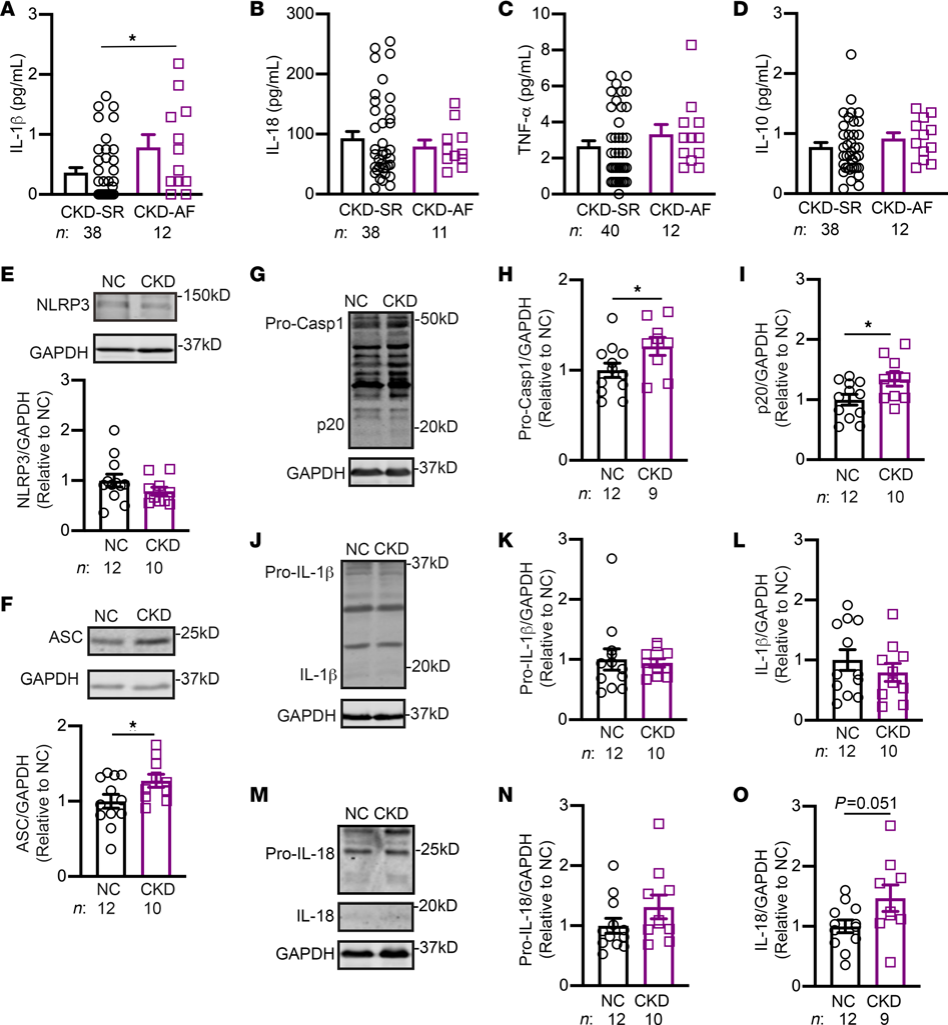
Enhanced NLRP3 inflammasomes activity in CKD patients with AF. Levels of the cytokines IL-1β (**A**), IL-18 (**B**), TNF-α (**C**), and IL-10 (**D**) in serum samples from dialysis-dependent patients with CKD-SR or CKD-AF. Representative Western blots and quantification of protein levels of NLRP3 (**E**) and ASC (**F**). Representative Western blots (**G**) and quantification of pro–caspase-1 (Pro-Casp1) (**H**) and mature caspase-1 (p20) (**I**). Representative Western blots (**J**) and quantification of pro–IL-1β (**K**) and mature IL-1β (**L**). Representative Western blots (**M**) and quantification of pro–IL-18 (**N**) and mature IL-18 (**O**). Data are expressed as the mean ± SEM. *P < 0.05, by unpaired 2-tailed Student’s *t* test (**A**, **F**, **H**, **I**, and **O**).

To determine whether AF development in patients with CKD was correlated with an enhanced inflammatory state, we performed a multiplex assay to examine the circulating levels of various cytokines in serum samples from a cohort of dialysis-dependent patients with CKD in sinus rhythm (CKD-SR) or AF (CKD-AF), respectively. Within the CKD-AF group, 5 patients had paroxysmal AF and 7 had persistent AF. Patient characteristics are provided in Supplemental Table 1 (supplemental material available online with this article; https://doi.org/10.1172/JCI167517DS1). Among the cytokines assayed, we found that only IL-1β was significantly elevated in patients with CKD-AF, while the levels of IL-18, TNF-α, and the antiinflammatory cytokine IL-10 were similar for the CKD-SR and CKD-AF groups (Figure 1, A–D). The levels of IL-1β were similar among patients with CKD with paroxysmal AF or persistent AF (Supplemental Figure 1). To determine whether the activity of the NLRP3 inflammasome was altered in atrial tissue of patients with CKD, we evaluated the levels of key proteins of the NLRP3 inflammasome pathway in a second cohort of patients undergoing open-heart surgery for coronary bypass grafting and/or valve replacement. Patients with an estimated glomerular filtration rate (eGFR) of 81 mL/min or higher were selected for the normal control (NC) group. Patients with an eGFR of 42mL/min or lower were selected for the CKD group. Patient characteristics are provided in Supplemental Table 2. Although protein levels of atrial NLRP3 were unchanged when comparing NC and CKD patients (Figure 1E), protein levels of ASC (Figure 1F), pro–caspase-1 (Figure 1, G and H), and cleaved caspase-1 (p20, Figure 1, G and I) were all significantly upregulated in atria of patients with CKD (*P* < 0.05 vs. NC). Full-length gasdermin-D (FL-GSDMD) was also upregulated in atria of patients with CKD (*P* < 0.01 vs. NC, Supplemental Figure 2). Surprisingly, protein levels of pro–IL-1β, mature IL-1β (Figure 1, J–L), and pro–IL-18 (Figure 1, M and N) were similar for NC and CKD groups; but protein levels of mature IL-18 trended toward increased expression in the CKD group (*P* = 0.051 vs. NC, Figure 1, M and O). Moreover, to determine whether the type of underlying heart disease affects activation of the NLRP3 inflammasome, we further analyzed the protein levels of inflammasome pathway mediators in atrial tissue samples from NC and CKD patients who underwent coronary artery bypass graft surgery (CABG) and valve replacement surgery. Within this small cohort of patients (Supplemental Table 2), protein levels of p20 and mature IL-18 were still significantly increased in atria of patients with CKD compared with NC patients (Supplemental Figure 3). These data suggest that the effect of CKD on atrial inflammatory signaling was robust and probably independent of the development of valvular heart disease. Overall, these results suggest that enhanced activation of the NLRP3 inflammasome correlates with AF development in patients with CKD.

### CKD activates the NLRP3 inflammasome in mouse atria.

**Figure 2:**
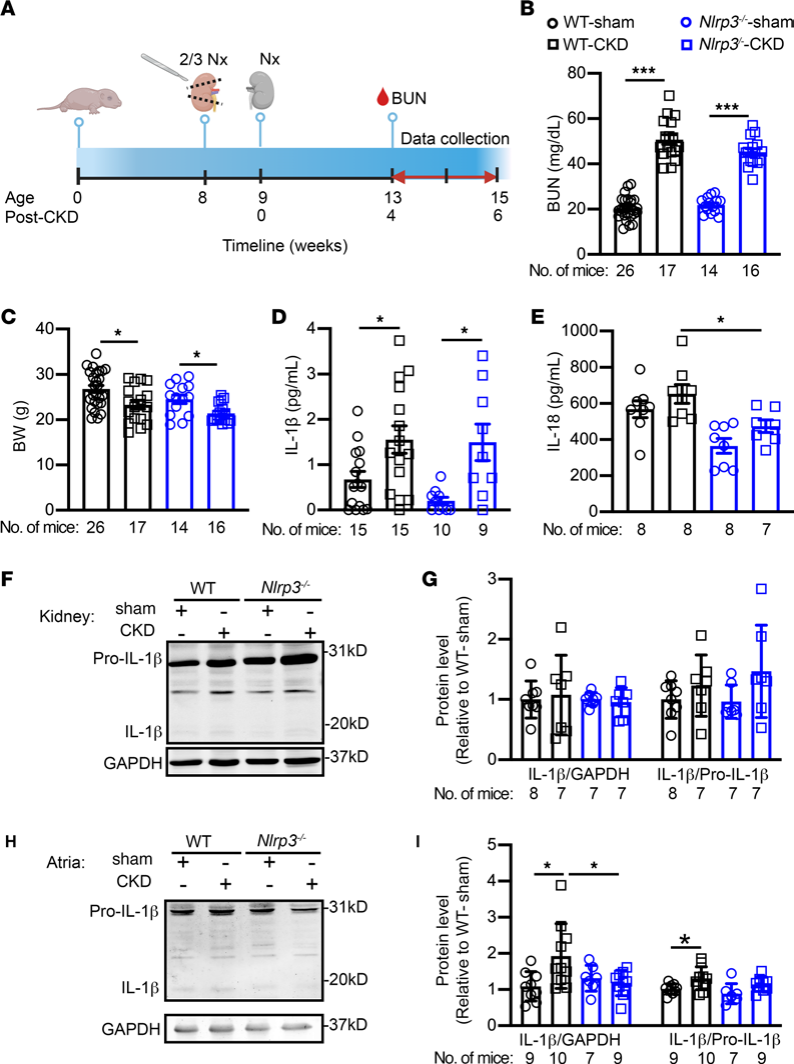
Murine model of CKD exhibits increased NLRP3 inflammasome activity. (**A**) Timeline of the 2-stage subtotal nephrectomy to generate the CKD mouse model. The schematic was created with BioRender.com. (**B**) BUN levels and (**C**) BWs in WT and *Nlrp3^–/–^* mice subjected to sham or CKD procedures. (**D** and **E**) Serum levels of IL-1β and IL-18. (**F**) Representative Western blots and (**G**) quantification of IL-1β in kidney tissue**.** (**H**) Representative Western blots and (**I**) quantification of IL-1β in atrial tissue. Data are expressed as the mean ± SEM. *P < 0.05 and ***P < 0.001, by Welch’s ANOVA and Dunnett’s T3 multiple-comparison test (**B** and **C**) and ordinary 1-way ANOVA with Šidák’s multiple-comparison test (**D** and **I**).

To determine whether kidney dysfunction upregulates the NLRP3 inflammasome complex in atria, we adopted the previously established 2-stage subtotal nephrectomy protocol (30, 31) to create a CKD model in WT (WT-CKD) and *Nlrp3*-knockout CKD (*Nlrp3^–/–^*-CKD) mice (Figure 2A). Sham-operated mice served as controls. Four weeks after the second nephrectomy (i.e., after CKD development), blood urea nitrogen level (BUN) levels were significantly increased in both WT-CKD and *Nlrp3^–/–^*-CKD mice compared with sham-operated mice, respectively (*P* < 0.001, Figure 2B). Additionally, compared with their respective sham controls, both WT-CKD and *Nlrp3^–/–^*-CKD mice exhibited a significant decrease in BW (*P* < 0.05, Figure 2C), indicating a loss of body mass due to kidney dysfunction. Both male and female mice exhibited similar patterns of changes in BUN levels and BWs (Supplemental Figure 4). Following the confirmation of CKD development, we performed ELISA to assess serum levels of IL-1β and IL-18 in mice. Consistent with the data obtained for patients with CKD, serum levels of IL-1β, but not IL-18, were increased in WT-CKD compared with WT sham-operated (WT-sham) mice (Figure 2D). To further demonstrate the link between CKD and the increased levels of circulating IL-1β cytokines, we measured IL-1β expression in bone marrow of WT-sham and WT-CKD mice, given that CKD can cause the mineral and bone disorder (32) and that bone marrow is a major source of hemopoietic cells and cytokine release. We found that *Il1b* was significantly upregulated in the bone marrow of WT-CKD mice (vs. WT-sham, *P* < 0.05) (Supplemental Figure 5). These results indicate that increased cytokine production in bone marrow of CKD mice may contribute to an increase in circulating IL-1β levels and subsequent activation of the NLRP3 inflammasome pathway in atrial tissue. Indeed, direct application of IL-1β cytokines on H9C2 cells (the rat myoblast cell line) increased NLRP3 expression and activity, leading to increased protein levels of mature IL-1β in H9C2 cells (Supplemental Figure 5).

Interestingly, global inhibition of NLRP3 significantly reduced circulating levels of IL-18, but not of IL-1β, in *Nlrp3^–/–^*-CKD versus WT-CKD mice (Figure 2, D and E). These data suggest that other forms of inflammasomes in other tissues may be activated as well, potentially contributing to the higher circulating IL-1β level following kidney injury. Moreover, in the remaining kidney tissue of WT-CKD mice, the levels of matured IL-1β were unchanged compared with levels in kidney tissues of WT-sham mice (Figure 2, F and G), indicating that more cleaved IL-1β was released into the blood in WT-CKD mice than in WT-sham mice. However, in atrial tissue, the levels of mature IL-1β were still significantly higher in WT-CKD mice than in WT-sham mice (*P* < 0.05, Figure 2, H and I). Compared with WT-CKD mice, levels of mature IL-1β were reduced in *Nlrp3^–/–^*-CKD mice (*P* < 0.05, Figure 2, H and I). These results suggest that CKD caused NLRP3inflammasome activation within the atria. Moreover, genetic inhibition of NLRP3 was sufficient to prevent the increased IL-1β cleavage in atria of *Nlrp3^–/–^*-CKD mice.

### CKD creates an atrial arrhythmic substrate via the NLRP3 pathway.

**Figure 3:**
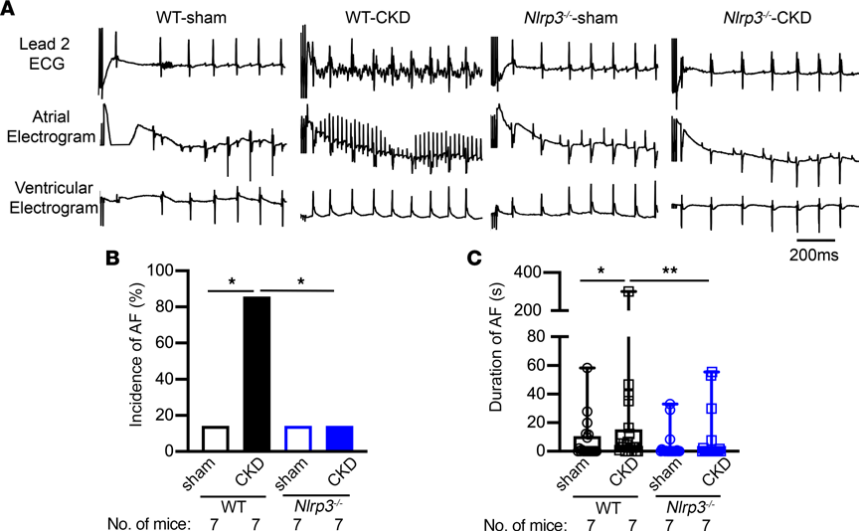
Increased AF susceptibility in CKD mice. (**A**) Representative recordings of lead 2 surface ECG and intracardiac atrial and ventricular electrograms in WT and *Nlrp3^–/–^* mice subjected to sham or CKD procedures. (**B**) Incidence of pacing-induced reproducible AF. (**C**) Duration of pacing-induced AF. Data are expressed as a percentage in **B** and as the mean ± SEM in **C**. *P < 0.05 and **P < 0.01, by Fisher’s exact test (**B**) and Kruskal-Wallis test with Dunn’s multiple-comparison test (**C**).

ECG parameters including heart rate (HR), PR-, QRS-, and QTc-intervals, sinus node recovery time (SNRT), atrioventricular Wenckebach (AVW), and atrioventricular node effective refractory period (AVNERP) 4 weeks after CKD surgery were similar in WT-sham, WT-CKD, *Nlrp3*^–/–^-sham, and *Nlrp3*^–/–^-CKD mice (Supplemental Table 3). To assess the AF susceptibility of these mice, we performed programmed electrical stimulation (PES) studies in the 4 groups of mice. While only 14.2% (1 or 7) of the WT-sham mice exhibited pacing-induced AF, 86% (6 of 7) of the WT-CKD mice developed pacing-induced AF (*P* < 0.05, Figure 3, A and B). Additionally, the average duration of all AF episodes was longer in the WT-CKD mice than in the WT-sham mice (*P* < 0.05, Figure 3C). In contrast, knockout of *Nlrp3* reduced both the incidence and duration of pacing-induced AF in *Nlrp3^–/–^*-CKD mice (*P* < 0.05, *P* < 0.01 vs. WT-CKD, Figure 3, A–C). These data reveal that CKD promoted the evolution of an arrhythmogenic substrate for AF, which required activation of the NLRP3 inflammasome pathway.

### CKD promotes electrical remolding via the NLRP3 pathway.

**Figure 4:**
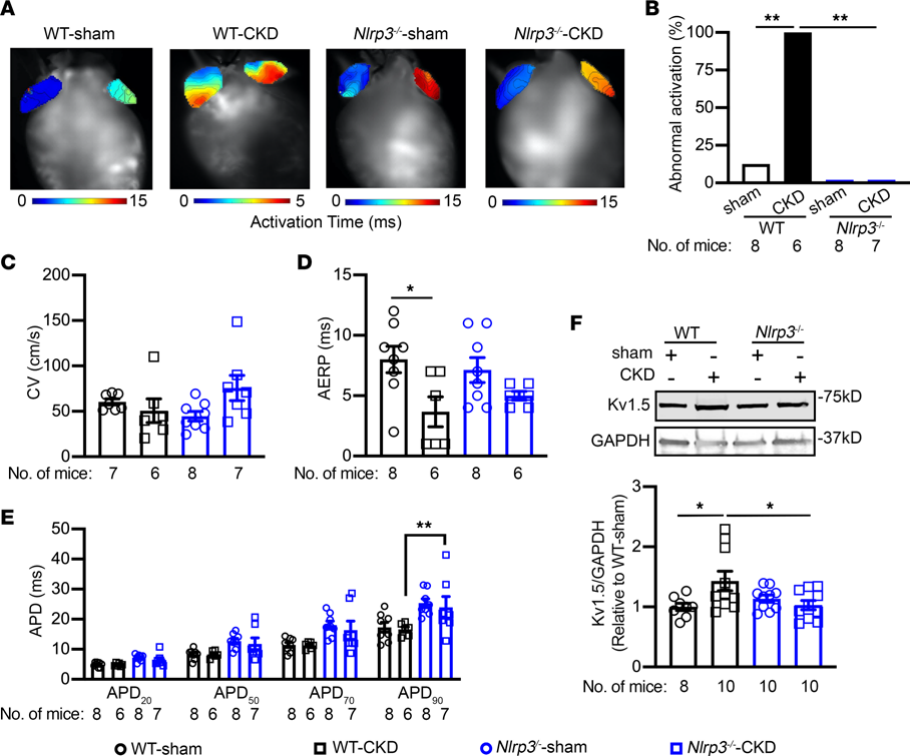
CKD promotes electrical remodeling for AF. (**A**) Representative activation maps from an optical mapping study. (**B**) Incidence of abnormal activation patterns in each treatment group. (**C** and **D**) Summary of CV (**C**) in the RA and AERP (**D**) in atria of WT and *Nlrp3^–/–^* mice subjected to sham or CKD procedures. (**E**) APD at 20%, 50%, 70%, and 90% repolarization. (**F**) Kv1.5 protein levels in atria of WT and *Nlrp3^–/–^* mice subjected to sham or CKD procedures. Data are expressed as a percentage in **B** and as the mean ± SEM in **C**–**F**. *P < 0.05 and **P < 0.01, by Fisher’s exact test (**B**), ordinary 1-way ANOVA with Šidák’s multiple-comparison test (**D** and **F**), and 2-way ANOVA with Tukey’s multiple-comparison test (**E**).

To determine the effect of CKD on activation patterns and conduction, we performed optical mapping studies. Interestingly, all WT-CKD mice displayed abnormal activation patterns, indicative of ectopic activity, compared with the WT-sham mice at a pacing cycle length (CL) of 100 ms, whereas none of the *Nlrp3^–/–^* mice exhibited abnormal activation patterns (Figure 4, A and B). While the conduction velocity (CV) in the right atrium (RA) was comparable among the 4 groups of mice (Figure 4C), the atrial effective refractory period (AERP) was significantly abbreviated in WT-CKD mice compared with WT-sham mice (Figure 4D). The AERP was not different when comparing *Nlrp3^–/–^*-sham and *Nlrp3^–/–^*-CKD mice (Figure 4D). The action potential duration at 90% repolarization (APD_90_) trended toward a decrease in the atria of WT-CKD compared with WT-sham mice and was reversed in *Nlrp3^–/–^*-CKD mice (*P* < 0.01, vs. WT-CKD, Figure 4E). These results suggest that CKD induced the development of electrical remodeling as a potential arrhythmogenic substrate for AF and that inhibition of NLRP3 prevented CKD-induced shortening of the APD and AERP. To further delineate the molecular determinants underlying the shortening of the AERP, we evaluated the expression of major ion channel subunits including the voltage-dependent Na^+^ channel (Nav1.5), the α-subunit of L-type Ca^2+^ channel (Cav1.2), and the ultra-rapid delayed rectifier K^+^ channel (Kv1.5) in atrial tissue of WT and *Nlrp3^–/–^* mice. We found that the protein levels of Nav1.5 and Cav1.2 were comparable among WT and *Nlrp3^–/–^* mice with sham surgery or CKD, respectively (Supplemental Figure 6). We have previously shown that constitutive activation of NLRP3 in a cardiomyocyte-specific knockin mouse model reduces the AERP by enhancing the expression and activity of Kv1.5 channels. Accordingly, Kv1.5 protein levels were upregulated in WT-CKD compared with WT-sham mice (*P* < 0.05, Figure 4F). On the other hand, Kv1.5 protein levels were normalized in *Nlrp3^–/–^*-CKD mice (*P* < 0.05, Figure 4F). These results implicate NLRP3-mediated Kv1.5 upregulation as a major contributor to electrical remodeling in mice with CKD-induced AF.

### CKD promotes structural remodeling and fibrosis via the NLRP3 pathway.

**Figure 5:**
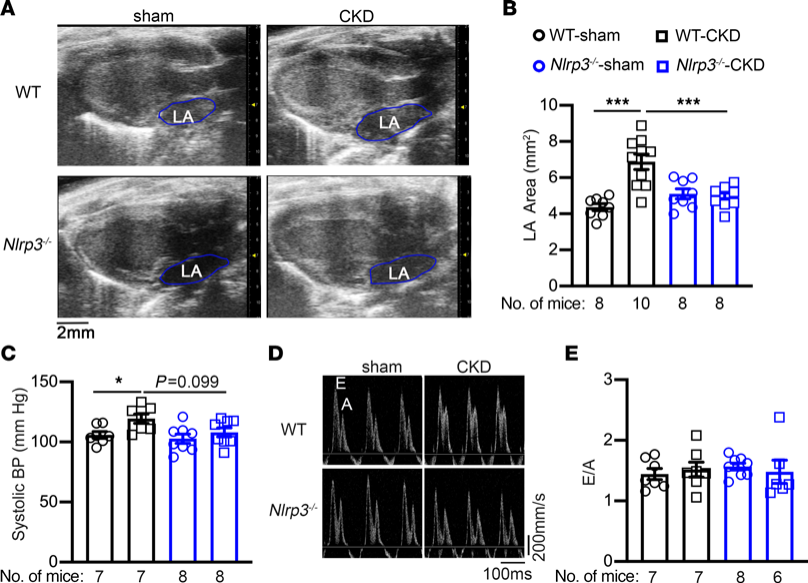
CKD promotes structural remodeling. (**A**) Representative echocardiographic images of the long-axis view of hearts. (**B**) Quantification LA areas in WT and *Nlrp3^–/–^* mice that were subjected to sham or CKD procedures. (**C**) SBP measurement. (**D**) Representative pulsed-wave Doppler images. (**E**) Quantification of E/A ratios. Data are expressed as the mean ± SEM**.** *P < 0.05 and ***P < 0.001, by ordinary 1-way ANOVA with Šidák’s multiple-comparison test (**B** and **C**).

To determine whether structural remodeling contributes to the enhanced AF inducibility seen in WT mice with CKD, we examined the dimensions of left atrium (LA) using echocardiography (Figure 5A). The LA area was significantly increased in WT-CKD mice compared with WT-sham mice (*P* < 0.001, Figure 5B). Inhibition of NLRP3 prevented the CKD-associated LA enlargement in *Nlrp3^–/–^*-CKD mice (*P* < 0.001 vs. WT-CKD, Figure 5, A and B). Because kidney function plays an integral part in modulating blood pressure (BP), which in turn affects atrial loading, we measured systolic blood pressure (SBP) using the tail-cuff method. Consistent with a previous report (33), WT-CKD mice exhibited a significantly increased SBP compared with WT-sham mice (*P* < 0.05, Figure 5C). Whole-body knockout of NLRP3 reduced SBP in *Nlrp3^–/–^*-CKD mice compared with WT-CKD mice (*P* = 0.099, Figure 5C). These results suggest that global inhibition of NLRP3 could normalize atrial size by improving the CKD-induced increase in afterload.

To evaluate whether the enhanced AF susceptibility in the CKD model was associated with ventricular dysfunction, we assessed the contractility and dimensions of the left ventricle using echocardiography. We found that all 4 groups of mice had a similar ejection fraction (EF%), left ventricular diameters (end-systolic diameter and end-diastolic diameter), and left ventricular wall thicknesses (Supplemental Figure 7 and Supplemental Table 4). Moreover, a pulse wave Doppler revealed that the early to later ventricular filling velocity (E/A) ratios were comparable among the 4 groups (Figure 5, D and E), suggesting that diastolic function was also preserved in CKD mice 4 weeks after the second nephrectomy. These data indicate that ventricular dysfunction did not contribute to enhanced AF susceptibility in the context of CKD.

**Figure 6:**
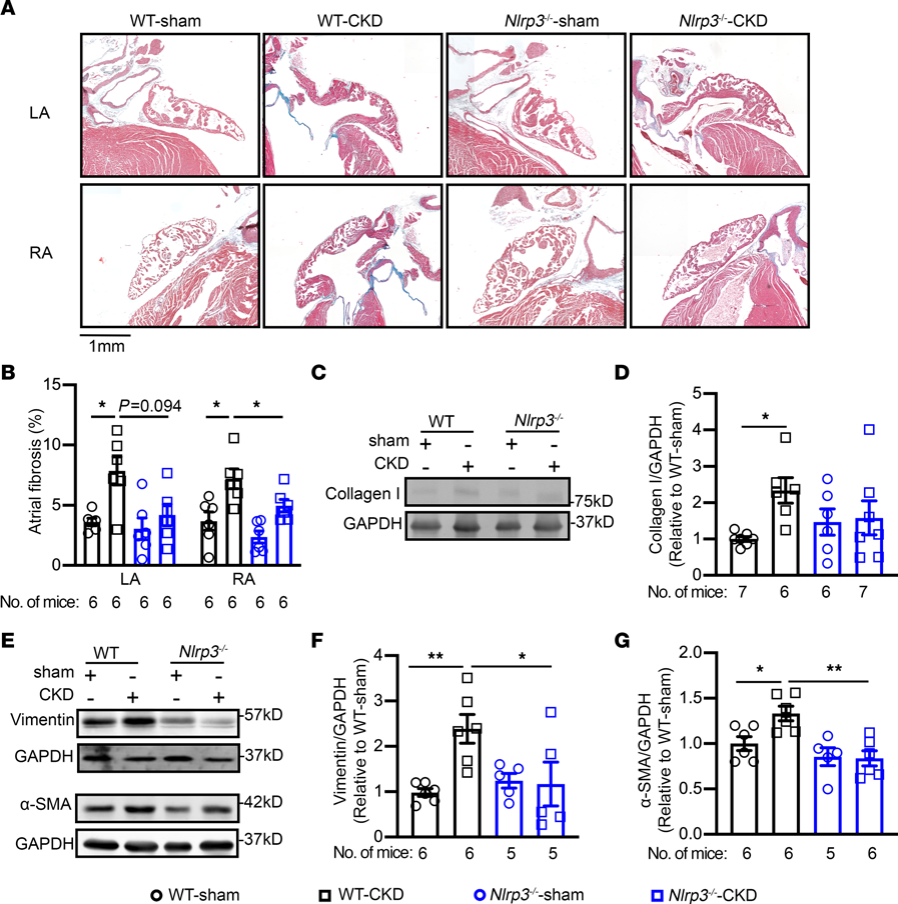
CKD promotes atrial fibrosis. (**A**) Representative images of Masson’s trichrome staining of heart sections from WT and *Nlrp3^–/–^* mice subjected to sham or CKD procedures. Scale bar: 1 mm. (**B**) Quantification of fibrosis in the LA and RA of WT and *Nlrp3^–/–^* mice subjected to sham or CKD procedures. (**C**–**G**) Representative Western blots and quantification of collagen I (**C** and **D**), vimentin (**E** and **F**), and α-SMA (**E** and **G**). Data are expressed as the mean ± SEM**.** *P < 0.05 and **P < 0.01, by Welch’s ANOVA and Dunnett’s T3 multiple-comparison test (**B**) and ordinary 1-way ANOVA with Šidák’s multiple-comparison test (**D**, **F**, and **G**).

Next, we examined whether CKD promotes atrial fibrosis, a known substrate for AF development. In the atrial tissue sections of patients, Picrosirius red staining revealed that fibrosis was higher in patients with CKD compared with NC patients (*P* < 0.05, Supplemental Figure 8). Consistently, Masson’s trichrome staining revealed that the percentage of fibrosis in the LA and RA were both similarly increased in WT-CKD mice compared with WT-sham mice (*P* < 0.01, Figure 6, A and B), whereas ventricular fibrosis was comparable among the 4 groups (Supplemental Figure 9). H&E staining and immune staining for F4/80 revealed that a mild increase in immune cell infiltration in atrial tissue of WT-CKD mice compared with WT-sham mice (Supplemental Figure 10). Consistent with the presence of atrial fibrosis, protein levels of fibrogenic markers including collagen I, vimentin, and α–smooth muscle actin (α-SMA) (Figure 6, C–G), but not collagen III (Supplemental Figure 9), were elevated in atria of WT-CKD versus WT-sham mice. In contrast, atrial fibrosis and increased protein levels of collagen I, vimentin, and α-SMA as well as increased atrial levels of F4/80 were all attenuated by inhibition of NLRP3 in *Nlrp3^–/–^*-CKD mice (Figure 6 and Supplemental Figure 10). These results suggest that enhanced NLRP3 signaling in CKD caused fibrotic remodeling that may have contributed to the atrial substrate-promoting arrhythmogenesis.

### Neutralization of IL-1β prevents structural remodeling and reduces AF inducibility in CKD.

**Figure 7:**
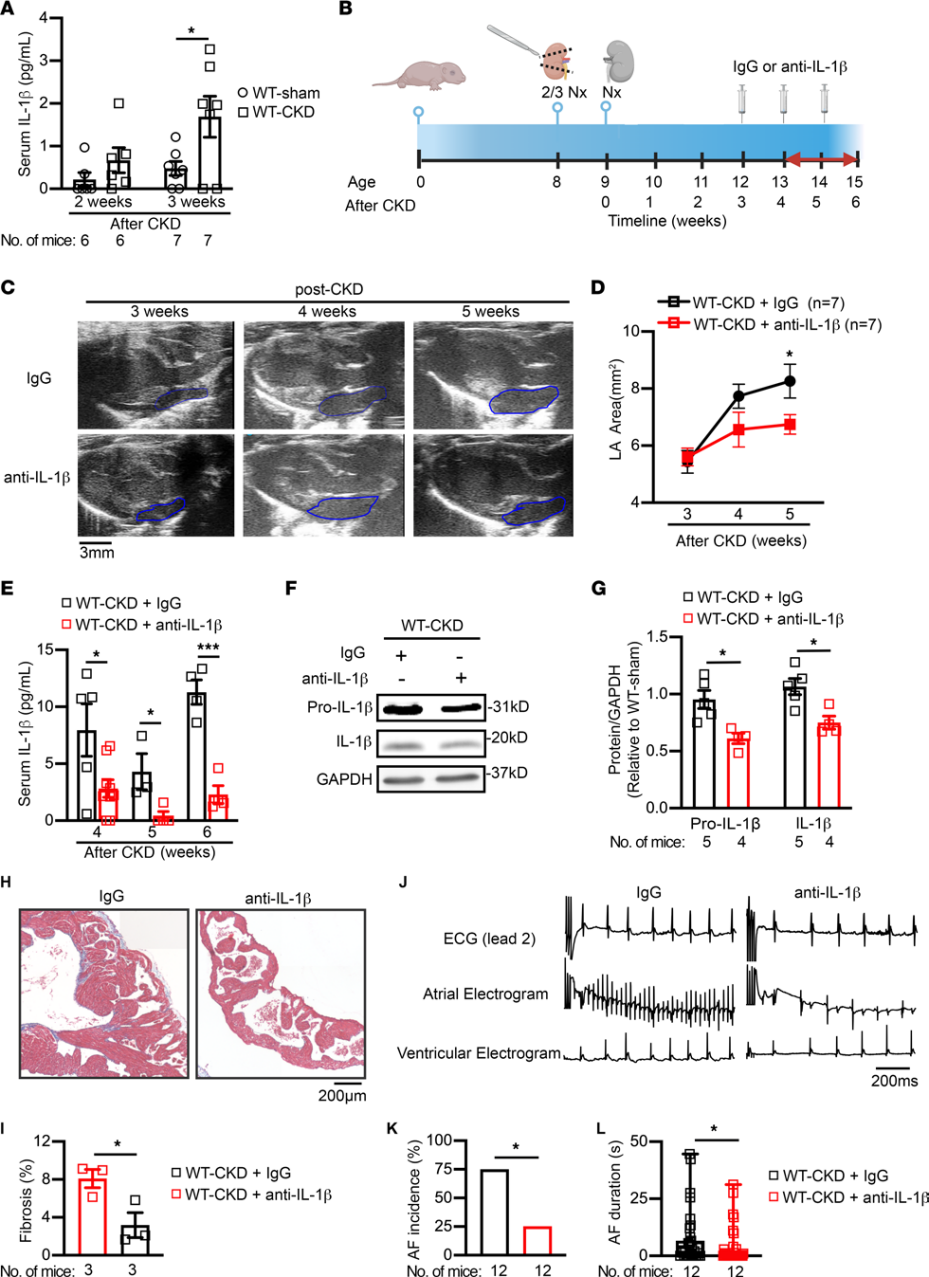
Neutralization of circulating IL-1β attenuates CKD-induced atrial arrhythmogenesis. (**A**) Serum levels of IL-1β in WT mice 2 and 3 weeks after sham or CKD surgeries. (**B**) Timeline of anti–IL-1β antibody (5 mg/kg, i.p.) or IgG (as a control) injections into WT-CKD mice. 2/3 Nx, two-thirds nephrectomy. Panel **B** was created with BioRender.com. (**C** and **D**) Representative echocardiographic long-axis views of the heart (**C**) and quantification of the LA area (**D**) in WT-CKD mice that received IgG or anti–IL-1β antibody injections. Blue outlined areas indicate the LA. Scale bar: 3 mm. (**E**) Serum levels of IL-1β in WT-CKD mice that received IgG or anti–IL-1β antibody injections following the CKD procedure. (**F** and **G**) Representative Western blots (**F**) and quantification (**G**) of IL-1β in atrial tissue of WT-CKD mice after 3 weekly injections of IgG or anti–IL-1β antibody. (**H** and **I**) Representative images (**H**) of Masson’s trichrome staining in atria and quantification of atrial fibrosis (**I**) in WT-CKD mice after 3 weekly injections of IgG or anti–IL-1β antibody. Scale bar: 200 μm. (**J**–**L**) Representative lead 2 surface ECG and intracardial ECG recordings (**J**), incidence (**K**), and duration (**L**) of pacing-induced AF in WT-CKD mice after 3 injections of IgG or anti–IL-1β antibody. Scale bar: 200 ms (**J**). Data are expressed as the mean ± SEM in **A**, **D**, **E**, **G**, **I**, and **L**, and as a percentage in **K.** *P < 0.05 and ***P < 0.001, by unpaired, 2-tailed Student’s *t* test (**A**, **D**, **E**, **G**, and **I**), Fisher’s exact test (**K**), and Mann-Whitney *U* test (**L**).

Because circulating IL-1β levels were increased in patients with CKD and mice with an increased susceptibility to AF induction, we also evaluated whether neutralization of circulating IL-1β could prevent the development of AF. First, we determined the time point when circulating IL-1β levels started to increase in our CKD mouse model. We found that, compared with age-matched WT-sham mice, serum IL-1β levels were significantly increased in WT-CKD mice 3 weeks, but not 2 weeks, after the second nephrectomy procedure (Figure 7A). Next, in a separate cohort of mice, we injected WT-CKD mice weekly with either anti–IL-1β antibody or IgG as a placebo (5 mg/kg, i.p.), starting 3 weeks after the second nephrectomy (Figure 7B). Echocardiography was performed before and weekly following the first injection. We found that ventricular function remained unchanged when comparing CKD mice receiving IgG with CKD mice receiving anti–IL-1β antibodies (Supplemental Table 5). Whereas the LA area progressively increased in WT-CKD mice treated with IgG, the WT-CKD mice injected with anti–IL-1β antibody exhibited a slower increase in the LA area (Figure 7, C and D). The difference in LA area between IgG- and anti–IL-1β–treated WT-CKD mice became statistically significant 2 weeks after injections (*P* < 0.05, Figure 7D). Serum levels of IL-1β were determined weekly following the first injection. Injections of anti–IL-1β antibody reduced circulating IL-1β levels (Figure 7E) but did not alter BUN levels (Supplemental Figure 11) in WT-CKD mice compared with IgG-treated mice. After 3 weekly injections, we performed a PES study and harvested tissues for Western blotting and histological analysis. We found that mature IL-1β levels in atrial tissue were reduced in WT-CKD mice treated with anti–IL-1β antibody compared with WT-CKD mice given the IgG placebo (Figure 7, F and G). Moreover, atrial fibrosis was attenuated in WT-CKD mice that received anti–IL-1β antibody compared with those that received the IgG placebo (Figure 7, H and I). Ultimately, the injections with anti–IL-1β antibody significantly reduced the incidence and duration of pacing-induced AF in WT-CKD compared with IgG-treated WT-CKD mice (Figure 7, J–L). These results demonstrate that IL-1β neutralization could effectively ameliorate atrial structural remodeling, thereby reducing atrial arrhythmogenesis associated with CKD.

## Discussion

CKD is a progressive disease that is associated with systemic inflammation. Both CKD and inflammation are well-known risk factors for cardiovascular disease (CVD) and cardiac arrhythmias. However, it remains unclear how CKD-induced inflammation leads to arrhythmogenesis. The present study demonstrates a mechanistic link between the NLRP3/IL-1β signaling axis and CKD-related AF development. We found increased IL-1β levels in the serum of dialysis-dependent patients with CKD with AF compared with those in sinus rhythm. Similarly, mice with CKD exhibited a higher susceptibility to AF, elevated IL-1β serum levels, and increased atrial expression of IL-1β, implicating atrial activation of NLRP3 as the key driver of AF inducibility. We further demonstrated that either suppression of NLRP3 or neutralization of IL-1β could prevent CKD-induced AF by reducing atrial fibrosis.

Patients with CKD have a greater risk of developing AF than do healthy individuals (6), especially among patients undergoing dialysis treatment (34). The combination of dialysis and AF has been related to an increased risk of death (35, 36). Because of this, several studies have assessed the potential mechanisms underlying AF development in CKD (34, 37, 38). Although several mechanisms, including augmented inflammation, myocardial fibrosis, and LA enlargement (39), have been proposed to contribute to AF development in patients with CKD, none of these has been addressed directly. Here, we found that IL-1β production was substantially higher in dialysis-dependent CKD patients with AF than in those in sinus rhythm. Pathological elevation of members of the IL-1 family has been associated with a greater risk of CVD. The involvement of IL-1β in inflammation-derived CVD is well known (40). For example, it has been shown that IL-1β plays a role in endothelial dysfunction, abdominal aortic aneurysm formation, and atherosclerotic plaques in humans (41, 42). Canakinumab Anti-inflammatory Thrombosis Outcome Study (CANTOS) demonstrated that inhibition of IL-1β in patients with atherosclerosis can reduce the incidence of major cardiovascular events (43, 44). Furthermore, IL-1β has also been shown to affect cardiac contractility and induce proarrhythmogenic changes in vitro and in vivo (45). In patients with heart failure symptoms, there is a correlation between circulating IL-1β levels and the incidence of AF (46). Similarly, in the current study, we found higher levels of circulating IL-1β to be associated with an increased incidence of AF in patients with CKD, further confirming a pathogenic role of IL-1β in the onset of AF. We also demonstrate a link between CKD and increased circulating levels of the cytokine IL-1β. Emerging evidence suggests that CKD promotes mineral bone disorders (32), which affect the function of bone marrow — the major source of hemopoietic stem cells and immune cells. We present evidence that IL-1β signaling was upregulated in the bone marrow of CKD mice, which could partially explain the systemic inflammatory status associated with the CKD development. This evidence opens a door for future investigations into the role of CKD-mediated hemopoietic dysfunction in CVD. More important, we show that neutralizing circulating IL-1β by treating mice with anti–IL-1β antibody could reduce the susceptibility to CKD-induced AF, providing a proof of concept for targeting IL-1β in patients with CKD who are at risk for AF.

The processing of pro–IL-1β into its mature form is mediated by the enzymatic activity of caspase-1, which in turn is activated by the NLRP3 inflammasome (47). We have previously shown that activation of NLRP3 in a cardiomyocyte-specific knock-in mouse model promotes the development of ectopic activity, electrical remodeling, and secondary fibrosis, which lead to increased AF inducibility (29). The current study demonstrates increased activity of NLRP3 in the atria of CKD mice, with increased IL-1β levels in atrial samples from WT-CKD mice. This is consistent with earlier work that reported an increase in NLRP3 activity in the atria of a CKD rat model (48). Inhibition of NLRP3 blunted the increase in IL-1β levels in atrial samples from *Nlrp3^–/–^* mice subjected to the same CKD procedures. Similarly, other groups have found increased NLRP3 expression in nephrectomized mice with increased levels of IL-1β (21, 22, 49). Various studies have shown that the subtotal nephrectomy model of CKD can cause contractile dysfunction with a reduced ejection fraction anywhere between 6 and 12 weeks after the second nephrectomy (22, 33, 50). Because heart failure can independently enhance the risk for AF and is often associated with inflammatory changes, we intentionally determined the AF inducibility at an earlier time point following the nephrectomy procedures to avoid confounding effects. The preserved normal ventricular function 5 weeks after the second nephrectomy in our CKD model helped us to confirm that inflammasome signaling was the primary cause of AF promotion in this CKD model. Indeed, our CKD mice developed abnormal atrial activation (suggestive of triggered activity), shortening of the AERP, and atrial fibrosis with atrial enlargement, all of which are well-known arrhythmogenic determinants of AF. Given the previous observations that NLRP3 activation is associated with an increased incidence of spontaneous Ca^2+^ release events in cardiomyocytes (25, 28, 29), we suspect that the onset of AF in CKD model could be driven by enhanced Ca^2+^ release–induced triggered activity. Additionally, our finding that CKD mice developed structural remodeling of the atria is consistent with prior findings in a rat model of CKD induced by either subtotal nephrectomy or an adenine-rich diet (48, 51). Several previous studies have suggested that activation of NLRP3 in cardiomyocytes and cardiac fibroblasts can increase the functional activity of fibroblasts via indirect and direct mechanisms, ultimately enhancing collagen production and causing structural remodeling (29, 52, 53). Enlarged atria and atrial fibrosis are expected to disturb atrial conduction. Therefore, along with AERP shortening, structural remodeling–related conduction disturbances can promote reentry circuits, thereby enhancing the AF inducibility associated with CKD. Together, these findings support the idea that CKD-induced systemic inflammatory factors activate the atrial NLRP3 inflammasome, thereby promoting the onset of AF. This hypothesis is supported by the fact that genetic ablation of NLRP3 protected against the abnormal atrial activation, atrial fibrosis, and atrial enlargement induced by CKD. Although circulatory levels of IL-1β were similar in WT and NLRP3-knockout CKD mice, the atrial expression levels of IL-1β were lower in the latter group, demonstrating reduced activation of the NLRP3 inflammasome pathway in this group of mice.

It is well known that the NLRP3 inflammasome is activated by DAMPs, which occur as a consequence of organ damage due to CKD. NLRP3 synthesis is promoted by the binding of IL-1 to its receptors, together with pro–IL-1β and pro–IL-18. Because no changes were observed in circulating IL-18 levels between CKD patients with and without AF or between WT-sham and WT-CKD mice, IL-1β is probably the main trigger of NLPR3 activation in the atria in CKD. This hypothesis is supported by our finding that neutralization of circulating IL-1β in CKD mice reduced atrial IL-1β levels, impeded the development of atrial fibrosis and atrial enlargement, and prevented the inducibility of AF in CKD. Similar results were reported by other studies, in which IL-1β blockade improved cardiac function and atherosclerosis-induced cardiac events (43). A recent study suggests that renal denervation can improve LA structural remodeling and reduce AF susceptibility in rats with CKD induced by an adenine-rich diet (51). Our study presents a noninvasive therapeutic approach to reduce the risk of AF in CKD by neutralizing anti–IL-1β antibodies.

Our study has some limitations. The levels of circulating cytokines and NLRP3 inflammasome proteins in atrial tissue were obtained from 2 independent cohorts of patients. We cannot draw direct correlation between circulating IL-1β levels and the activity of NLRP3 in atria of patients with CKD. We were unable to obtain a sufficient number of atrial biopsy samples from patients with CKD and AF. Therefore, we could not determine whether the activity of the atrial NLRP3 inflammasome was further enhanced in CKD patients with AF compared with the CKD patients with sinus rhythm. Given the invasive nature of the 2-stage nephrectomy procedure, we could not implant ECG telemeters into these mice to monitor ambulatory ECG readings. Thus, we could not assess the incidence or duration of spontaneous atrial arrhythmias in this CKD model. Other nonsurgical CKD models could be adapted in future studies to determine the impact of CKD on AF burden.

In conclusion, this study demonstrates that the proinflammatory milieu induced by CKD enhanced atrial NLRP3 inflammasome activity, caused atrial electrical remodeling and fibrosis, and increased AF inducibility. Our data position IL-1β blockade or, potentially, direct inhibition of NLRP3 as a pharmacological intervention to prevent AF development in patients with CKD.

## Methods

### Animal model of CKD.

All mice were housed under 12-hour light/12-hour dark cycles. In cases in which anesthesia was required, mice were anesthetized by inhaled 1.5%–2% isoflurane (Henry Schein Animal Health) in 100% oxygen (0.8–1.2 L/min). *Nlrp3* homozygous knockout mice (*Nlrp3^–/–^*) were purchased from The Jackson Laboratory (stock no. 017969) and backcrossed with C57BL/6J mice for more than 6 generations (25). Age- and sex-matched C57BL/6J WT and *Nlrp3^–/–^* mice underwent subtotal nephrectomy in 2 stages. During the first stage, two-thirds of the left kidney was removed. One week later, the entire right kidney was removed (30, 54). Mice were fed a normal chow diet before and after the procedures. Sham-treated control mice underwent surgery without damaging the kidneys and were fed the same diet. Four weeks after the second nephrectomy, BUN levels were measured in all 4 groups of mice: (a) WT-sham, (b) WT-CKD, (c) *Nlrp3^–/–^*-sham, and (d) *Nlrp3^–/–^*-CKD. For the IL-1β neutralization studies, 3 weeks after the second nephrectomy, WT-CKD mice were injected weekly with anti–IL-1β antibody (Thermo Fisher Scientific, catalog 16-7012-38) or lgG as a control (Thermo Fisher Scientific, catalog 16-4888-81) for 3 weeks.

### Echocardiography.

Briefly, mice were anesthetized using 1.5%–2% isoflurane mixed with 100% O_2_ and placed on a heated platform, where 4 limbs were taped to ECG electrodes. Body temperature was monitored via a rectal temperature probe and was kept at 37°C. Systolic function of the left ventricle and LA size were assessed with the VisualSonics Vevo 2100 (VisualSonics) (55).

### PES.

The PES technique was performed 1–2 weeks after BUN levels were measured (29). Mice were anesthetized using 1.5%–2.0% isoflurane in 100% O_2_. A 1.1 F octapolar catheter (EPR-100, Millar Instruments) was inserted into the heart by cannulating the right jugular vein. Bipolar RA pacing was administered via an external stimulator (STG-3008, MultiChannel Systems). Electrophysiological data for SNRT and AVERP were obtained using standard clinical pacing protocols. AF susceptibility was determined using a rapid atrial burst pacing protocol as described previously (25, 29). The experimenter was blinded to the mouse genotype and/or treatment group.

### Optical mapping.

Hearts were dissected and cannulated via the aorta and retrograde perfused and superfused with Tyrode’s solution (2–5 mL/min). Di4-ANEPPS (Invitrogen, Thermo Fisher Scientific, 0.1 mmol/L, 1 mL) was slowly injected into a drug port to load the heart with the voltage-sensitive dye. To eliminate motion artifacts, blebbistatin (MilliporeSigma, 6.8 mmol/L, 0.1 mL) was delivered to the heart via the drug port. The emission Vm-signal was long-passed (>700 nm) and acquired via a MiCAM CMOS camera (SciMedia) at a sampling rate of 1 kHz and a pixel size of 100 mm/pixel. RA pacing and ex vivo ECG were recorded by a PowerLab 26 T stimulator (AD Instruments). The AERP was assessed with S1–S2 extrastimuli (CL, 100 ms) during a pulse train (pulse width, 5 ms; pulse amplitude, 5 V). ElectroMap was used to generate the activation map and analyze conduction velocity and APD at 20%, 50%, 70%, and 90% repolarization. For each value, the average of 10 consecutive beats at 10 Hz pacing was calculated for each mouse.

### Western blotting.

Heart samples were dissected and flash-frozen in liquid nitrogen. Protein samples were separated on acrylamide gels, transferred onto PVDF membranes at 4°C followed by a 1-hour block, and subsequently probed with antibodies. Antibodies used for the mouse samples are listed in Supplemental Table 6. Densitometry was analyzed by ImageJ (NIH).

### ELISA.

Serum was collected from WT and *Nlrp3^–/–^* mice subjected to the sham or CKD procedures, respectively. IL-1β and IL- 18 were determined following the instruction of the ELISA kits (Mouse IL-1β/IL-1F2 Quantikine ELISA Kit, R&D Systems, MLB00C; Mouse IL-18 ELISA kit, Medical and Biological Laboratories, code no. 7625).

### Histologic analysis.

Whole hearts were dissected from the anesthetized mice, perfused with 4% KCl, and then fixed in 4% paraformaldehyde for 48 hours, prior to embedment in paraffin. Sections (6 mm thick) were collected and subjected to Masson’s trichrome or H&E staining. Images were acquired using a Zeiss microscope. The percentage of fibrosis in tissue sections was quantified using ImageJ.

### Statistics.

Numerical data are presented as the mean ± SEM. A 2-tailed Student’s *t* test was used to compare data between 2 groups of data with normal distribution. A Mann-Whitney *U* test was used to compare nonparametric equivalent of the 2-group samples without normal distribution. ANOVA followed by Šidák’s or Dunnett’s T3 multiple-comparison test was used for multiple comparisons. A Kruskal-Wallis test with Dunn’s post hoc test was used to compare nonparametric data with multiple comparisons. Fisher’s exact test was used to compare categorical data. A *P* value of less than 0.05 was considered statistically significant.

### Study approval.

For the assessment of cytokine levels in patients with CKD, serum samples were collected from patients undergoing dialysis. All experimental protocols were approved by the human ethics committee of the Hospital Universitario in Madrid, Spain (CEI: 16/250) and were performed in accordance with the Declaration of Helsinki. Written informed consent was obtained from all patients. Deidentified samples were provided to the Cardiorenal Translational Laboratory for the assessment of biomarkers in blood samples. Patient characteristics are listed in Supplemental Table 1. For Western blotting analysis of human atrial samples, RA appendage samples were collected from patients undergoing open-heart surgery for CABG and/or valve replacement. The experimental protocols were approved by the human ethics committee of the Medical Faculty of the University Duisburg-Essen (approval no. AZ:12-5268-BO) and were performed in accordance with the Declaration of Helsinki. Written informed consent was obtained from all patients. Patient demographics and characteristics are listed in Supplemental Table 2. All studies involving mice were approved by the IACUC of the Baylor College of Medicine (protocol no. AN-7259, AN-8213) and conformed to the *Guide for the Care and Use of Laboratory Animals* published by the NIH (National Academis Press, 2011).

### Data availability.

The detailed experimental materials, methods, and data supporting the findings of this study are available within the article and its supplemental material. Values for all data points in graphs can be found in the Supplemental Supporting Data Values file.

## Author contributions

NL, XHTW, JS, and JANG designed the research studies. JS, JANG, JW, AS, IAT, LL, SKL, JAK, YAS, OMM, YY, XW, and GRH conducted experiments and acquired and analyzed data. WEM, SST, ZH directed JW on establishing the CKD mouse model. MK collected RA appendage samples from patients. JS and JANG drafted the manuscript and shared responsibilities as co–first authors. The order of the co–first authors’s names was determined by workload. DD oversaw the experiments and data analysis in human atrial samples and critically revised the figures and the manuscript. NL and XHTW critically revised the figures and the manuscript and shared responsibilities as senior authors.

## Supplementary Material

Supplemental data

Supporting data values

## Version 1 08/15/2023

In-Press Preview

## Version 2 10/02/2023

Electronic publication
