# Supplementary material for: Chronic kidney disease promotes atrial fibrillation via inflammasome pathway activation
Source: J Clin Invest. 2023 Oct 2;133(19):e167517. doi: 10.1172/JCI167517 (PMC10541185; doi:10.1172/JCI167517)
Supplement: Supplemental data [file jci-133-167517-s065.pdf]

Chronic kidney disease promotes atrial fibrillation via inflammasome pathway activation

Jia Song, Jose Alberto Navarro-Garcia, Jiao Wu, Arnela Saljic, Issam Abu-Taha, Luge Li, Satadru K Lahiri, Joshua A Keefe, Yuriana Aguilar-Sanchez, Oliver M. Moore, Yue Yuan, Xiaolei Wang, Markus Kamler, William E. Mitch, Gema Ruiz-Hurtado, Zhaoyong Hu, Sandhya S. Thomas, Dobromir Dobrev, Xander H.T. Wehrens, Na Li

Supplementary Methodology

Human Subjects. Cohort 1: To assess the inflammatory cytokine levels in CKD patients, serum samples were collected from patients undergoing dialysis treatment. All experimental protocols were approved by the Human Ethics Committee of the Hospital Universitario 12 de Octubre in Madrid, Spain (CEI: 16/250, Spain) and were performed in accordance with the Declaration of Helsinki. Written informed consent was obtained from all patients. De-identified samples were provided to the Cardiorenal Translational Laboratory for assessment of biomarkers in blood samples. Patient characteristics in **Supplementary Table S1**. A total of 52 patients (46.2% men) from the Dialysis Unit of the Hospital Universatario 12 de Octubre in Madrid were included in the study. Average age of the patients was 63.4 years. Blood samples were collected in EDTA tubes before initiating dialysis procedure. Cytokines were measured by PorcartaPlex Immunoassay (ThermoFisher, Cat# PPX-04). Cohort 2: To assess the protein levels of NLRP3 inflammasome and fibrosis in atria of CKD patients, right atrial appendage samples were collected from patients undergoing open-heart surgery for coronary bypass grafting and/or valve replacement. Patients with the estimated glomerular filtration rate (eGFR) ≥81mL/min were selected as normal control (NC) group. Patients with eGFR ≤42mL/min were selected as CKD group. All experimental protocols were approved by the Human Ethics Committee of the Medical Faculty of the University Duisburg-Essen (approval number AZ:12-5268-BO) and were performed in accordance with

the Declaration of Helsinki. Written informed consent was obtained from all patients. Patient demographics and characteristics are listed in **Supplementary Table S2.**

Electrophysiology. Programmed intracardiac stimulation (PIS) was performed to establish cardiac electrophysiological differences. Briefly, a 1.1F octapolar electrode catheter (EPR-800, Millar Instruments, Houston, TX) was inserted into the right atria and right ventricle via the right jugular vein. A 2-second burst pacing protocol starting at a cycle length (CL) of 40-ms and decreasing in each successive burst by a 2-ms decrement to a CL of 10-ms was performed to determine AF inducibility. AF was defined as the occurrence of rapid, fragmented atrial electrograms with irregular R-R intervals lasting more than 1-second. A mouse was considered positive for AF if it had at least 2 AF episodes out of 3 induction attempts. The percentage of positive mice in each group was calculated as the AF inducibility. The experimenter was blinded to genotype or treatment status of mice.

Systolic blood pressure measurements. A noninvasive blood pressure analysis system (BP-2000, Visitech Systems, Apex, NC) was used to measure indirect blood pressure and heart rate for conscious non-anesthetized mice using tail cuff method.

Western Blotting. Protein was extracted by adding RIPA butter with protease and phosphatase inhibitors to the atrial and kidney samples on ice. Western blot analysis was performed using atrial and kidney lysates (60-100 μg). Protein samples were separated on 12.5% polyacrylamide gels for an hour, transferred onto PVDF membranes overnight at 4°C, and subsequently blocking was done with 5% BSA in TBS buffer for an hour in room temperature. Primary antibodies (**Supplementary Table S5**) were added to the membrane and incubated overnight at 4°C. Membranes were washed 3 times for 10 minutes each time with TBST., followed by incubation for 2-3 hours at 4°C with fluorescent-linked secondary antibodies (anti-mouse or anti-rabbit antibodies was used according to the species of the primary antibodies). Afterwards, membranes were washed 3 times for 10 minutes each time with TBST. Images

were performed using the Odyssey system (Li-COR Biosciences). Densitometry was analyzed by Image J.

Quantitative PCR (qPCR). RNA was extracted from the bone marrow tissue using the Trizol reagent (Invitrogen). 500 ng of total RNA was used for reverse transcription with a final reaction volume of 20 μL containing 4μL iScript Reverse transcription Supermix (Bio-Rad 1708841). Following reverse transcription, the cDNA diluted five-fold. Quantitative PCR was performed in triplicate for 40 cycles using 2.5 μl of the volume of the cDNA in a total volume of 10 μL that included 5 μL of PowerUP SYBR Green Master Mix (Thermo Fisher A25742). 1 μM forward and reverse primer was used in the reaction. mRNA was quantified by real-time PCR analysis using the QuantStudio5 (Thermo Fisher). Thermocycler conditions were 40 cycles of denaturation at 95℃ for 15 seconds and annealing and extension step of 60℃ for 60 seconds. The ΔCT method was used to quantify all relative mRNA levels using *Gapdh* as the reference and internal standard. All primers used for qPCR are listed in Supplementary Table S7.

ELISA. Serum samples were collected from WT and *Nlrp3*$^{-/-}$ mice subjected to a sham or CKD procedure, respectively. IL-1β and IL-18 were determined according to the manufacturer's instruction manual for the ELISA kits (Mouse IL-1 beta/IL1F2 Quantikine ELISA Kit, R&D Systems, MLB00C; Mouse IL-18 ELISA kit, Medical& Biological Laboratories, CODE No.7625).

IL-1β treatment of cells. H9C2 cardiomyocytes (obtained from ATCC) were cultured in DMEM-medium (Gibco) with 10% FBS and 1% penicillin/streptomycin at 37°C with 5% CO_2. Cells were treated with 50 ng/mL recombinant rat IL-1β (R&D Systems, 501RL) or PBS as control for 48h. Cells were extracted with RIPA and stored at -80°C.

Histology. Whole hearts were dissected from the anesthetized mice, perfused with 4% KCl, and then fixed in 4% paraformaldehyde for 48 hours, prior to embedding in paraffin. 6 μm sections were collected

and subjected to Hematoxylin & Eosin (HE) and Masson's trichrome staining. Images were acquired using a Zeiss microscope (Axio Observer 7). The percentage of fibrosis in tissue sections was quantified using FIJI. The region of interest was outlined with Polygon selection tool and was saved in ROI manager tool. Under the setting of RGB stack type, threshold was adjusted to reflect total tissue area (including both bule and red area) and the fibrosis area (blue-only area). The threshold level was consistent in each image. The percentage of fibrosis was calculated as the ratio of fibrosis area to total tissue area.

Immunohistochemical staining. Immunohistochemistry was conducted on paraffin embedded whole heart sections prepared as stated above. Paraffin-embedded sections were first hydrated through a series of washes in xylene (3x5 minutes), 100% ethanol (2x3 minutes), 95% ethanol (1 minute), 80% ethanol (1 minute), and dH2O (2x5 minutes). Sections were then heated at 95C for 10 minutes in citrate antigen retrieval buffer (10 mM citrate, 0.05% Tween-20, pH 6.0), followed by cooling at room temperature for 20 minutes. Sections were then incubated with 3% hydrogen peroxide for 10 minutes at room temperature, followed by blocking with 5% normal goat serum in TBST for 1 hour at room temperature. Anti-F4/80 antibody (Cell Signaling catalogue D2S9R, 1:500) was applied to the sections overnight at 4C. For each slide, a negative control section was incubated in blocking buffer. Slides were washed in TBST (3x5 minutes), followed by 30 minutes of incubation with boost detection reagent (Cell Signaling #8114). Slides were then exposed to DAB (3% SignalStain DAB Chromogen concentrate in SignalStain DAB Diluent, Cell Signaling #8059) for 2 minutes or until adequate staining intensity was achieved, followed by immersion into dH2O. Slides were then dehydrated by washes in 95% ethanol (2x1 minute), 100% ethanol (2x1 minute), and xylene (2x1 minute). Slides were then mounted using mounting medium (Cell Signaling #14177) and imaged. Quantification was performed using FIJI by thresholding each image according to the negative control to remove background.

Statistical analysis. Numerical data were presented as the mean ± SEM. Two-tailed Student's t-tests and 2-way-ANOVA were used to compare data between two groups with normal distributed values. Mann-Whitney test is used to compare nonparametric equivalent of the two-group samples without normal distribution. ANOVA followed by Sidak's or Dunnett's T3 multiple comparison tests were used for

multiple comparisons. Kruskal–Wallis test with post-hoc Dunn tests were used to compare nonparametric data with multiple comparisons. Fisher's exact test was used to compare categorical data. P-value of less than 0.05 was considered statistically significant.

Supplementary Tables

Table S1. Characteristics of the patients receiving dialysis treatment (Cohort 1).

	**CKD-SR**	**CKD-AF**	***p*-value**
Number of patients	40	12	
Age (years)	62.7 ± 15.5	65.8 ± 12.1	0.527
Sex (male, %)	23 (57.5)	6 (50)	0.746
BMI (kg/m^2)	23.5 ± 3.4	23.4 ± 3.5	0.963
SBP (mmHg)	128.6 ± 20.9	121.4 ± 27.6	0.471
DBP (mmHg)	72.0 ± 13.4	68.8 ± 14.3	0.471
HR (bpm)	75.3 ± 11.8	76.6 ± 12.8	0.795
**Medical history**			
Paroxysmal AF (%)	0 (0)	7 (58.3)	0.0001
Persistent AF (%)	0 (0)	5 (41.6)	0.0003
Hypertension (%)	32 (80)	11 (91.7)	0.666
LVH (%)	28 (70)	8 (66.7)	0.999
Dialysis vintage (years)	6.3 ± 5.1	14.8 ± 13.1	0.014
Calcium (mg/dL)	8.9 ± 0.6	9.1 ± 0.3	0.296
Phosphate (mg/dL)	4.3 ± 1.1	4.5 ± 1.6	0.712
**Medication**			
Anticoagulant (%)	20 (50)	7 (63.6)	0.508
Beta-blockers (%)	15 (37.5)	4 (36.4)	0.999
Anti-hypertensive (%)	11 (27.5)	2 (18.2)	0.706
Diuretics (%)	9 (22.5)	2 (18.2)	0.999
Anti-arrhythmics (%)	1 (2.5)	2 (18.2)	0.114

Data are presented as mean ± SD. CKD-SR: chronic kidney disease patients in sinus rhythm; CKD-AF: chronic kidney disease patients in persistent atrial fibrillation; BMI: body mass index; SBP: systolic blood pressure; DBP: diastolic blood pressure; HR: heart rate; LVH: left ventricular hypertrophy.

Table S2. Characteristics of patients undergoing open-heart surgery (Cohort 2)

	NC	CKD	*p*-value
Patients (n)	12	10	
Age (years)	61.3 ± 11.41	66.4 ± 9.40	0.275
Sex (female, %)	5 (41.7)	4 (40.0)	0.696
BMI (kg/m^2)	28.6 ± 4.98	29.8 ± 4.02	0.438
LVEF (%)	53.8 ± 7.27	51.0 ± 11.48	0.785
LA size (mm)	39.1 ± 6.45	43.2 ± 6.94	0.206
eGFR (mL/min)	94.1 ± 8.88	26.4 ± 12.54	<0.0001
Creatinine before surgery (μmol/L)	0.8 ± 0.05	2.6 ± 1.20	<0.0001
CRP before surgery (mg/L)	1.0 ± 1.77	1.7 ± 1.73	0.206
CRP after surgery (mg/L)	16.6 ± 7.61	18.0 ± 6.24	0.652
**Medical history**			
Hypertension (%)	10 (83.3)	10 (100.0)	0.176
Diabetes (%)	4 (33.3)	5 (50.0)	0.429
Dyslipidemia (%)	7 (58.3)	8 (80.0)	0.277
GABG (%)	8 (66.7)	5 (50.0)	0.429
AVD (%)	1 (8.3)	0 (0.0)	0.350
MVD (%)	0 (0.0)	1 (10.0)	0.262
CABG+AVD/MVD (%)	3 (25.0)	4 (40.0)	0.452
**Medication**			
Digitalis (%)	0 (0.0)	0 (0.0)	N/A
Beta-blockers (%)	5 (41.7)	8 (80.0)	0.069
ACE-inhibitors (%)	3 (25.0)	4 (40.0)	0.452
AT1-blockers (%)	3 (25.0)	2 (20.0)	0.780
Diuretics (%)	2(16.7)	5 (50)	0.095
Ca^{2+}-antagonists (%)	1 (8.3)	3 (30.0)	0.104
Lipid-lowering drugs (%)	7 (58.3)	8 (80.0)	0.277

Data are presented as mean ± SD. NC: normal kidney function patients in sinus rhythm; CKD: chronic kidney disease patients in sinus rhythm; BMI: body mass index; LVEF: Left ventricular ejection fraction; eGFR: estimated glomerular filtration rate(eGFR); CABG: Coronary artery bypass graft surgery; AVD, aortic valve disease; MVD, mitral valve disease; ACE, angiotensin converting enzyme.

Table S3. ECG parameters in wildtype (WT) and *Nlrp3*$^{-/-}$ mice

	WT-sham (n=7)	WT-CKD (n=7)	*Nlrp3*$^{-/-}$-sham (n=7)	*Nlrp3*$^{-/-}$-CKD (n=7)
HR (bpm)	525 ± 26	522 ± 13	475 ± 10	485 ± 17
PR (ms)	37.3 ± 1.53	35.2 ± 1.29	36.6 ± 0.67	39.4 ± 1.31
QRS (ms)	9.1 ± 0.18	9.6 ± 0.55	9.1 ± 0.47	9.3 ± 0.41
QTc (ms)	17.6 ± 0.90	17.8 ± 0.72	16.5 ± 0.66	18.5 ± 0.70
cSNRT100	1.44 ± 0.16	1.20 ± 0.08	1.02 ± 0.06	0.94 ± 0.09
cSNRT90	1.4 ± 0.16	1.2 ± 0.09	1.1 ± 0.10	0.95 ± 0.07
AVW	74.1 ± 2.87	70.3 ± 1.74	73.1 ± 1.01	71.3 ± 0.99
AVNERP100	52.8 ± 3.26	49.7 ± 1.20	46.4 ± 0.40	50.6 ± 2.64

HR, heart rate; SNRT, sinus node recovery time; AVW, atrioventricular Wenckebach; AVNERP, atrioventricular node effective refractory period. ANOVA followed by Dunnett's T3 multiple comparison tests were used for multiple comparisons. Kruskal–Wallis test with post-hoc Dunn tests were used to compare nonparametric data with multiple comparisons was used in here. Significance was not found between groups.

Table S4. Echocardiography parameters in WT and *Nlrp3*$^{-/-}$ mice 5-week following 2nd nephrectomy of sham or CKD procedures

	WT-sham (n=8)	WT-CKD (n=10)	*Nlrp3*$^{-/-}$-sham (n=8)	*Nlrp3*$^{-/-}$-CKD (n=8)
EF (%)	67.3 ± 0.83	68.9 ± 0.55	67.7 ± 1.23	70.8 ± 1.30
CO (mL/min)	21.2 ± 1.43	18.8 ± 1.10	19.6 ± 0.84	21.9 ± 1.26
ESD (mm)	2.39 ± 0.06	2.19 ± 0.06	2.38 ± 0.06	2.29 ± 0.06
EDD (mm)	3.78 ± 0.09	3.52 ± 0.09	3.77 ± 0.06	3.79 ± 0.06
LVAWs (mm)	1.01 ± 0.08	0.97 ± 0.06	1.09 ± 0.08	1.21 ± 0.03*
LVAWd (mm)	0.65 ± 0.05	0.59 ± 0.02	0.65 ± 0.06	0.71 ± 0.03
LVPWs (mm)	1.10 ± 0.08	1.00 ± 0.06	1.06 ± 0.06	1.15 ± 0.04
LVPWd (mm)	0.67 ± 0.03	0.60 ± 0.03	0.65 ± 0.04	0.70 ± 0.03

EF, ejection fraction; CO, cardiac output; ESD, left-ventricular end-systolic diameter; EDD, left-ventricular end-diastolic diameter; LVAWd, left-ventricular end-diastolic anterior wall thickness; LVAWs, left-ventricular end-systolic anterior wall thickness; LVPWd, left-ventricular end-diastolic posterior wall thickness; LVPWs, left-ventricular end-systolic posterior wall thickness. *$P<0.05$ WT-CKD vs *Nlrp3*$^{-/-}$-CKD by ANOVA with Sidak's multiple comparison tests.

Table S5. Echocardiography parameters in WT mice following CKD surgeries and injections of IgG or anti-IL-1β antibody

Post-CKD	3 weeks		4 weeks		5 weeks	
Injections	Baseline		1-week after injection		2-weeks after injections	
	IgG (n=7)	anti-IL-1β (n=7)	IgG (n=7)	anti-IL-1β (n=7)	IgG (n=7)	anti-IL-1β (n=7)
EF (%)	61.8 ± 0.81	62.9 ± 0.94	60.6 ± 1.83	61.0 ± 1.74	59.0 ± 1.32	61.05 ± 1.93
CO (mL/min)	24.2 ± 1.87	23.9 ± 1.37	26.9 ± 1.40	24.0 ± 1.27	27.7 ± 1.19	25.0 ± 1.67
ESD (mm)	2.76 ± 0.07	2.70 ± 0.12	2.95 ± 0.07	2.82 ± 0.14	3.01 ± 0.07	2.80 ± 0.13
EDD (mm)	4.11 ± 0.12	4.06 ± 0.16	4.36± 0.07	4.16 ± 0.15	4.37 ± 0.08	4.14 ± 0.14
LVAWs (mm)	0.82 ± 0.03	0.78 ± 0.03	0.86 ± 0.03	0.81 ± 0.02	0.86 ± 0.02	0.87 ± 0.02
LVAWd (mm)	0.53 ± 0.02	0.48 ± 0.02	0.56 ± 0.03	0.52 ± 0.03	0.60 ± 0.02	0.57 ± 0.03
LVPWs (mm)	0.85 ± 0.03	0.83 ± 0.02	0.93 ± 0.02	0.86 ± 0.03	0.96 ± 0.02	0.90 ± 0.02
LVPWd (mm)	0.55 ± 0.04	0.48 ± 0.02	0.59 ± 0.02	0.58 ± 0.02	0.60 ± 0.02	0.61 ± 0.03

EF, ejection fraction; CO, cardiac output; ESD, left-ventricular end-systolic diameter; EDD, left-ventricular end-diastolic diameter; LVAWd, left-ventricular end-diastolic anterior wall thickness; LVAWs, left-ventricular end-systolic anterior wall thickness; LVPWd, left-ventricular end-diastolic posterior wall thickness; LVPWs, left-ventricular end-systolic posterior wall thickness.

Table S6. Antibodies used in the study.

Target	Company and Catalogue	Dilution Rate
Primary antibody:		
NLRP3 (in human samples)	Novus Biologicals, NBP1-77080	1:1,000
ASC (in human samples)	Santa Cruz, sc-514414	1:1,000
Caspase-1 (in human samples)	Santa Cruz, sc-392736	1:1,000
NLRP3 (D4D8T) (in H9C2 cells)	Cell Signaling Technology,15101	1:1,000
ASC	Santa Cruz, sc-22514	1: 1,000
Caspase-1	Santa Cruz, sc-56036	1: 500
IL-1β	Abcam, ab9722	1:1,000
IL-18	Santa Cruz, sc-133127	1: 500
GSDMD	SIGMA, G7422	1: 1,000
Vimentin	Santa Cruz, sc-6260	1: 500
α-SMA	Abcam, ab124964	1: 10,000
Anti-Collagen III	Abcam, ab7778	1: 5,000
Anti-Collagen I	Santa Cruz, sc-59772	1:500
F4/80 (D2S9R) (Used for WB)	Cell Signaling Technology, 70076	1:1000
CD68	Abcam, ab201973	1:1000
Kv1.5	Novus Biologicals, NBP-76939	1:1000
Cav1.2	Alomone Labs, ACC-003	1:1000
Nav1.5	Alomone Labs, ACC-005	1:1000
GAPDH(D4C6R)	Cell Signaling Technology, 97166	1: 5,000
GAPDH (D16H11) XP(R)	Cell Signaling Technology, 5174	1: 5,000
F4/80 (D2S9R) (used for IHC)	Cell Signaling Technology, D2S9R	1:500
Secondary Antibody:		
Goat anti-Rabbit IgG(H+L) Alexa Fluor^{TM} Plus 800	Invitrogen, A32735	1:7,500
Goat anti-Mouse IgG(H+L) Alexa Fluor^{TM} Plus 680	Invitrogen, A32729	1:7,500
Goat anti-Rabbit IgG(H+L) Alexa Fluor^{TM} Plus 680	Invitrogen, A32734	1:7,500
Goat anti-Mouse IgG(H+L) Alexa Fluor^{TM} Plus 800	Invitrogen, A32730	1:7,500

Table S7. Primers used in this study.

Gene	Sequence (5'->3')
Il1b Fwd	TGCAGTGGTTCGAGGCCTAAT
Il1b Rev	GTGACCACTCTCCAGTACCCAC
Il18 Fwd	GTGAACCCCAGACCAGACTG
Il18 Rev	CCTGGAACACGTTTCTGAAAGA
Il6 Fwd	TGTGCAATGGCAATTCTGAT
Il6 Rev	GGTACTCCAGAAGACCAGAGGA
Gapdh Fwd	CGTCCCGTAGACAAAATGGT
Gapdh Rev	TTGATGGCAACAATCTCCAC

Supplementary Figures

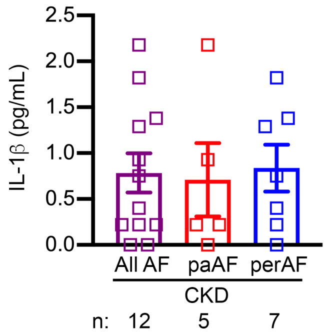

Figure S1. Similar levels of IL-1β cytokine in serum samples of paroxysmal AF (paAF) and persistent AF (perAF) patients with CKD.

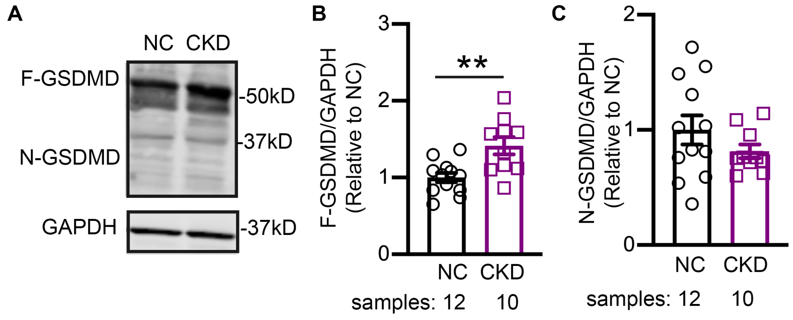

Figure S2. Enhanced NLRP3 inflammasomes activity in CKD patients with AF. Representative Western blots (**A**) and quantification of full length GSDMD (FL-GSDMD, **B**) and cleaved N-terminal GSDMD (Nt-GSDMD, **C**). ***P*<0.01*. P-value was determined by unpaired two-tailed Student's t-test in* ***B.***

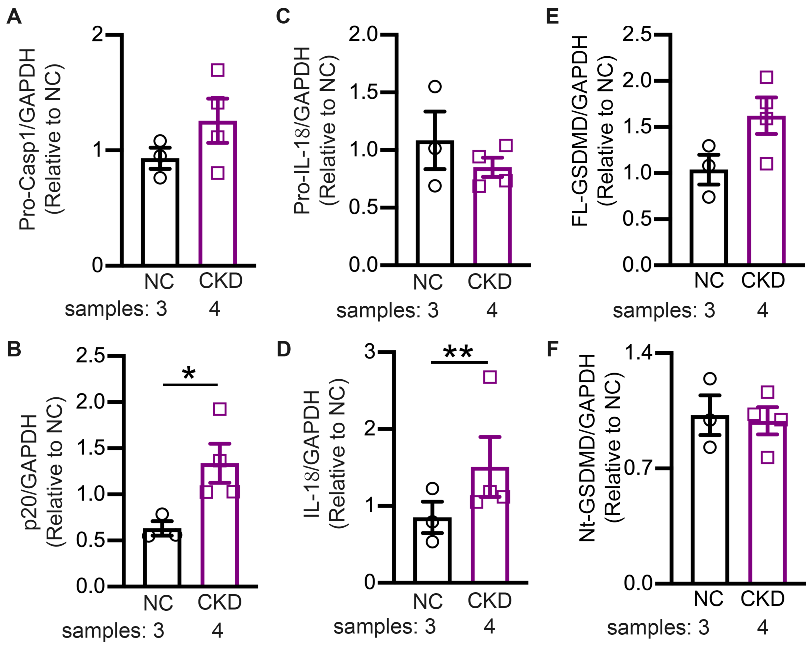

Figure S3. NLRP3 inflammasome was activated in CKD patients independent of valvular heart disease. Sub-analysis of Western blots results in right atrial appendage samples of normal control (NC) or CKD patients who underwent CABG and valve replacement surgery. Quantification of Pro-Casp1 (**A**) and p20 (**B**). Quantification of Pro-IL-18 (**C**) and IL-18 (**D**). Quantification of full length GSDMD (FL-GSDMD, **E**) and cleaved N-terminal GSDMD (Nt-GSDMD, **F**). *P<0.05, **P<0.01. *P-value was determined by 2-way-ANOVA in **B** and **D**.*

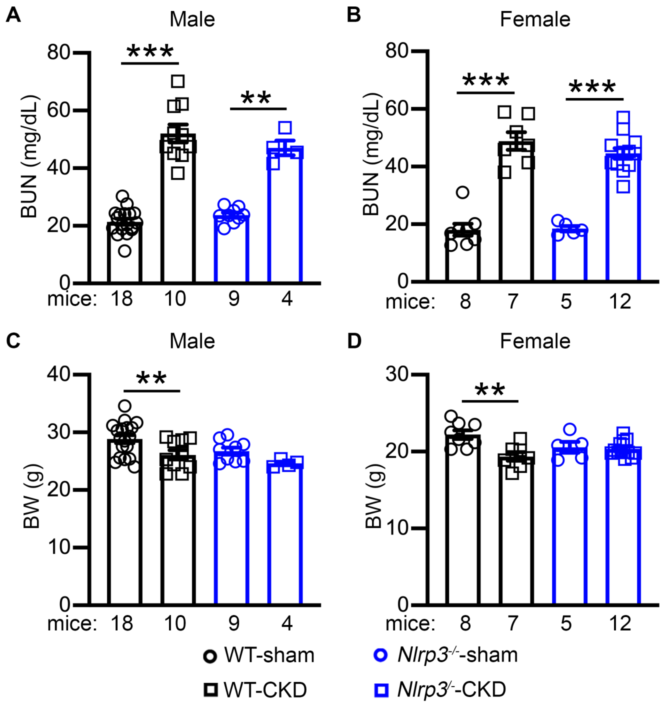

Figure S4. Similar patten of changes in BUN and BW in male(A, C) and female (B, D) of WT and *Nlrp3*$^{-/-}$ mice with sham or CKD respectively. **P<0.01, ***P<0.001. *P-value was determined by Welch ANOVA and Dunnett's T3 multiple comparison test.*

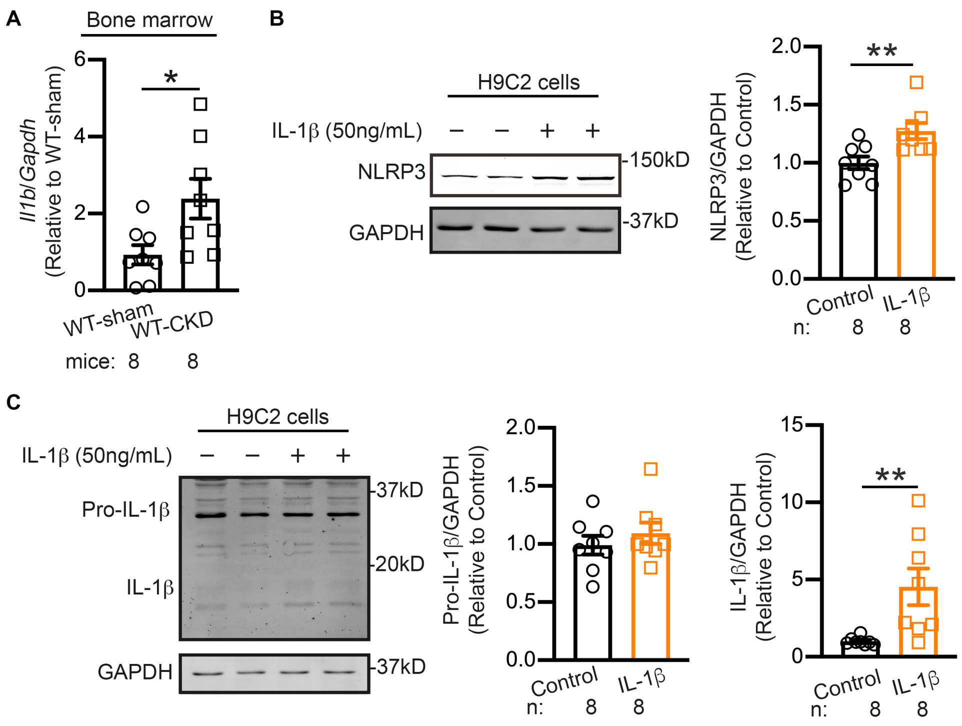

Figure S5. Elevated IL-1β level in bone marrow of WT-CKD mice and IL-1β-induced activation of NLRP3 in H9C2 cells. (**A**) Relative level of *Il1b* mRNA in bone marrow of WT-sham or WT-CKD mice. (**B-C**) Representative Western blots and quantification of NLRP3 (**B**) and Pro-IL-1β and mature IL-1β (**C**) in H9C2 cells treated with vehicle (control) or IL-1β cytokine for 48 hours. **P*<0.05*,* ***P*<0.01*. P-value was determined by unpaired two-tailed Student's t-test in* ***A, B*** *and* ***C.***

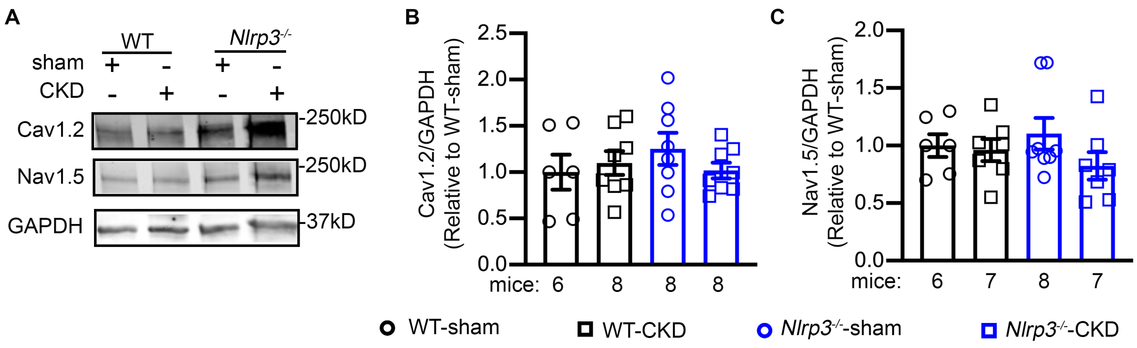

Figure S6. Unaltered protein level of alpha-subunit of L-type Ca^{2+} channel (Cav1.2) and voltage-dependent Na+ channel (Nav1.5) in CKD mice. (A) Representative Western blots and quantification of **(B)** Cav1.2 protein level and quantification of **(C)** Nav1.5 protein level in atrial tissues from WT and *Nlrp3*$^{-/-}$ mice with sham or CKD respectively.

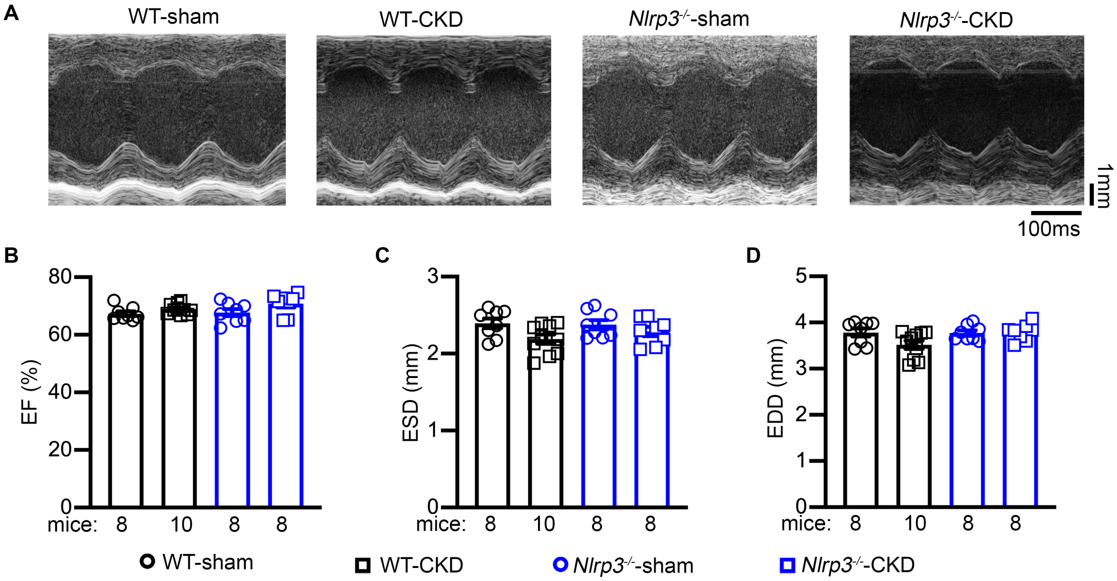

Figure S7. Unchanged systolic function. (**A**) Representative echocardiography images of M-mode short axis view of left ventricle. Quantification of EF% (**B**), end-systolic diameter (ESD, **C**), and end-diastolic diameter (EDD, **E**).

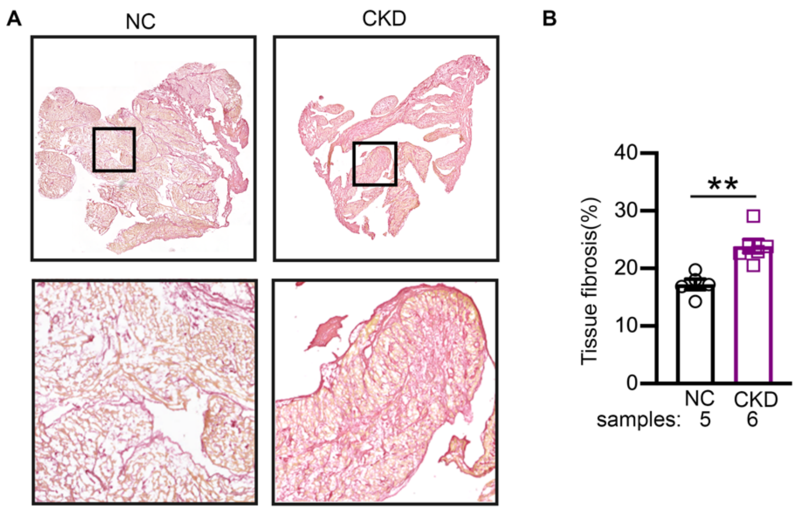

Figure S8. Increased tissue fibrosis in atrial sections of CKD patients. (A) Representative Picrosirius red staining in atrial tissue sections of normal control (NC) and CKD patients. **(B)** The quantification of fibrosis areas in atrial tissue of NC and CKD patients respectively. *P-value was determined by unpaired two-tailed Student's t-test in* ***B.***

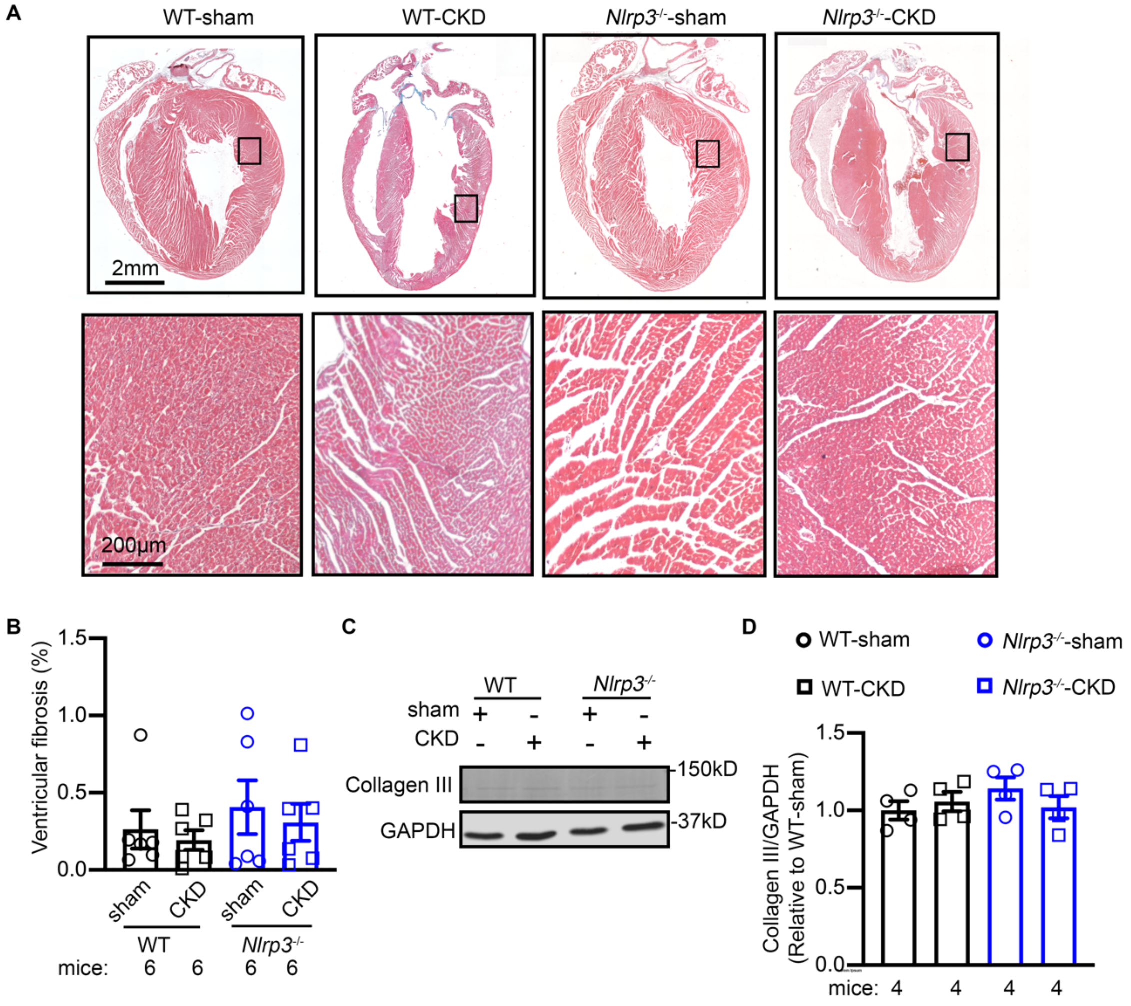

Figure S9. Unchanged ventricular fibrosis and collagen III in CKD mice. (**A**) Representative Masson's trichrome staining in apex of the ventricle. (**B**) The quantification of fibrosis areas in ventricles of WT and *Nlrp3*$^{-/-}$ mice with sham or CKD respectively. (**C-D**) Representative western blots (**C**) and quantification of collagen III in atrial tissue of WT and *Nlrp3*$^{-/-}$ mice with sham or CKD respectively (**D**).

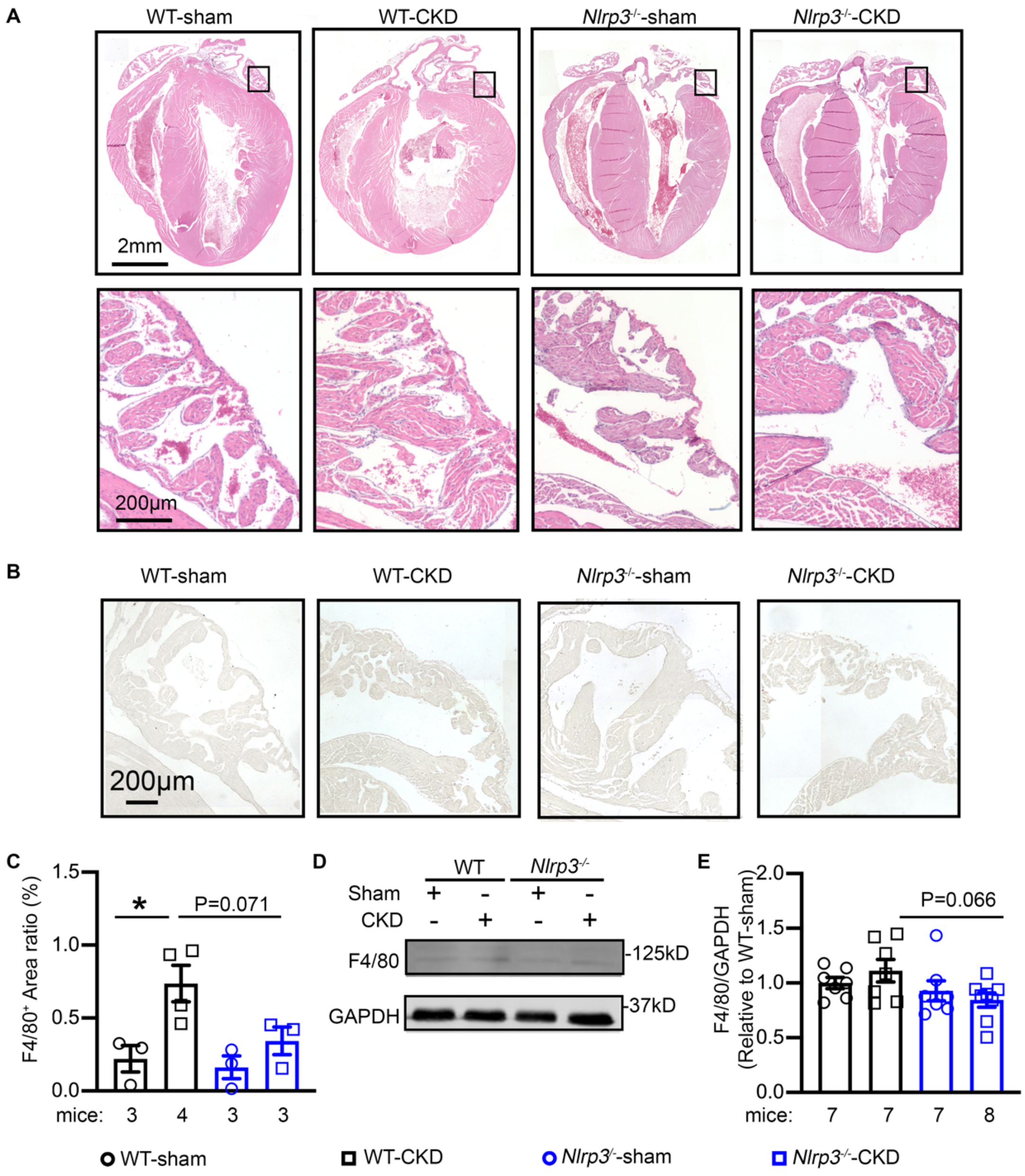

Figure S10. Mildly increased immune cell infiltration in WT-HPD group. (A) Representative images of Hematoxylin and eosin (H&E) staining in atrial tissue of WT and *Nlrp3*$^{-/-}$ mice with sham or CKD procedures, respectively. (**B**) Representative images and (**C**) quantification of immunohistochemical staining of F4/80 in atrial tissue in four groups. (**D**) Representative Western blots and (**E**) quantification of F4/80 protein level in atrial tissues from WT and *Nlrp3*$^{-/-}$ mice with sham or CKD respectively. *P-value was determined by* ordinary one-way ANOVA with Sidak's multiple comparison test in ***C*** and ***E.***

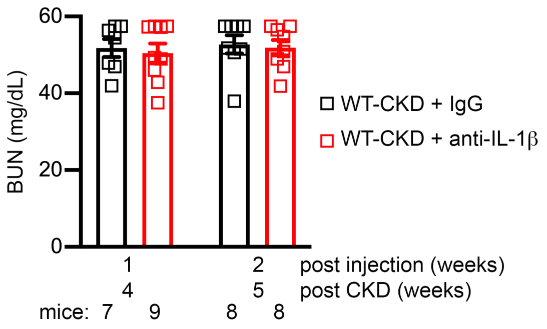

Figure S11. Unchanged blood urea nitrogen (BUN) levels in CKD model following IL-1β neutralization.
